# Dissociable encoding of motivated behavior by parallel thalamo-striatal projections

**DOI:** 10.1101/2023.07.07.548113

**Published:** 2023-07-07

**Authors:** Sofia Beas, Isbah Khan, Claire Gao, Emma McDonnald, Alison Bashford, Shakira Rodriguez-Gonzalez, Mario A. Penzo

**Affiliations:** 1 – Unit on the Neurobiology of Affective Memory, National Institute of Mental Health, Bethesda, MD, USA.; 2 – Department of Neurobiology, University of Alabama at Birmingham, Birmingham, AL, USA.

## Abstract

The successful pursuit of goals requires the coordinated execution and termination of actions that lead to positive outcomes. This process is thought to rely on motivational states that are guided by internal drivers, such as hunger or fear. However, the mechanisms by which the brain tracks motivational states to shape instrumental actions are not fully understood. The paraventricular nucleus of the thalamus (PVT) is a midline thalamic nucleus that shapes motivated behaviors via its projections to the nucleus accumbens (NAc)^1–9^ and monitors internal state via interoceptive inputs from the hypothalamus and brainstem^10–14^. Recent studies have revealed two major PVT neuronal subpopulations, Type1^PVT^ and Type2^PVT^, which differ in genetic identity, functionality, and anatomical connectivity to other brain regions, including the NAc^15–17^. In this study, we used fiber photometry to investigate the *in vivo* dynamics of these two distinct PVT neuronal types in mice performing a reward foraging task. We discovered that Type1^PVT^ and Type2^PVT^ neurons encode the execution and termination of goal-oriented actions, respectively. Furthermore, unlike Type2^PVT^ cells, activity in the Type1^PVT^ neuronal population mirrored motivation parameters such as vigor and satiety. Interestingly, these features were largely preserved when activity in PVT projections to the NAc was selectively assessed. Collectively, our results highlight the existence of two parallel thalamo-striatal projections that participate in the dynamic regulation of goal pursuits and provide insight into the mechanisms by which the brain tracks motivational states to shape instrumental actions.

## RESULTS

### Characterization of motivated behavior in rodents using a foraging-like task

Goal-oriented behaviors are generated by cortico-mesolimbic circuits that establish goal objects based on need states, as well as the actions required to obtain those objects^18^. Recent reports suggest that the PVT plays a critical role in translating needs states into motivation, owing to its strong innervation by the hypothalamus and brainstem^8,10,12–14^. The PVT is composed of molecularly diverse neuronal subpopulations that segregate across the anteroposterior axis of the thalamus and project to the NAc^16,17,19,20^. Particularly, the PVT contains two major distinct subpopulations, termed Type1^PVT^, and Type2^PVT^, respectively identified based on the expression of or lack thereof the dopamine D2 receptor^10,15–17^. However, how activity within parallel thalamo-striatal projections arising in the PVT relates to specific aspects of motivated behaviors remains unclear.

To address this question, we investigated the *in vivo* dynamics of Type1^PVT^ and Type2^PVT^ neurons using a foraging-like reward task^10,21^. In this task, mice initiated individual trials by entering a “trigger zone” where they were presented with a cue that signaled reward availability. Upon presentation of the cue, mice were required to shuttle to a “reward zone” to obtain a food reward (Supp. Fig. 1A) (See Methods). We found that mice successfully learned to shuttle back and forth to obtain the food reward and were highly engaged in the task, as demonstrated by two observations. First, they completed a significantly larger volume of trials in late compared to early sessions (Supp. Fig. 1B). Second, they displayed shorter latencies to reach the reward zone (Supp. Fig. 1C–E), obtain the reward (Supp. Fig. 1F–H), and initiate subsequent trials (Supp. Fig. 1I–K).

We noticed that mice varied in latencies to perform individual trials, but after training, they completed most trials under 11 seconds (~ 75% of completed trials; Supp. Fig. 1L). Thus, trial engagement was denoted by trials completed in 11 seconds or less. As such, to investigate how molecularly distinct subpopulations of the PVT encode various parameters associated with goal pursuit, the analysis of calcium signals recorded via fiber photometry was restricted to trials meeting the engagement criterion (Supp. Fig. 1 L–N). Importantly, this trial inclusion criteria allowed us to have a range of trials with different reward zone and reward delivery latencies that did not significantly differ throughout testing sessions (Supp. Fig. 1M–N) (See Methods). Moreover, it provided a suitable time frame for investigating the activity of Type1^PVT^ and Type2^PVT^ neurons during different trial groups and during different stages of the trials.

### Type1^PVT^ neuronal activity dynamics during food-seeking behavior

**Figure 1.**
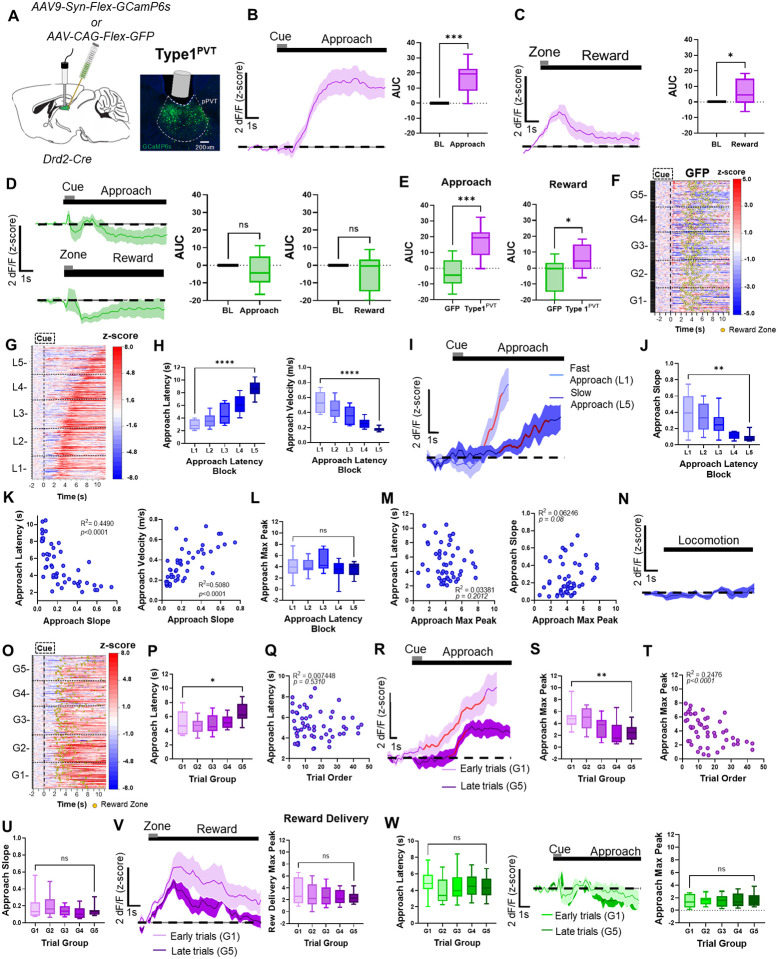
*In-vivo* activity dynamics of Type1^PVT^ neurons. **(A)** *Left*: Schematic of the stereotaxic injections and optical fiber implantation for targeting Type1^PVT^ neurons in the pPVT. *Right*: Representative image from a Drd2-Cre mouse expressing GCaMP6s in the pPVT. **(B)** *Left*: Average GCaMP6s responses from Type1^PVT^ neurons showing ramping activity during reward approach. *Right*: Quantification of the approach-evoked changes in GCaMP6s fluorescence in Type1^PVT^ neurons. Area under the curve (AUC), n = 296 trials from 6 mice, two-tailed paired t-test, ***p<0.001. **(C)** *Left*: Average GCaMP6s responses from Type1^PVT^ neurons during reward delivery. *Right*: AUC quantification of the reward-evoked changes in GCaMP6s fluorescence in Type1^PVT^ neurons. n = 296 trials from 6 mice, two-tailed paired t-test, *p<0.05. **(D)** *Left*: Top - Average GFP fluorescence during reward approach. Bottom - Average GFP activity during reward delivery. *Middle*: AUC quantification of GFP fluorescence during the baseline and approach periods. Two-tailed paired t-test, *p=0.29*; ns, not significant. *Right*: AUC quantification of GFP fluorescence during the baseline and reward delivery periods. Two-tailed paired t-test, *p=0.18*; ns, not significant. **(E)** *Left*: AUC quantification between GFP and Type1^PVT^ neuronal activity during reward approach. Two-tailed unpaired t-test, n = 544 trials from 5 mice, ***p<0.001. Right: AUC quantification between GFP and Type1^PVT^ neuronal activity during reward delivery. AUC, Two-tailed unpaired t-test, *p<0.05. **(F)** Heatmap of GFP activity in the PVT, time-locked to cue onset, sorted by trial order and binned into 5 ‘trial group blocks’ (G1 – G5). Yellow dots represent reward zone arrival. **(G)** Heatmap showing excitatory reward approach responses from Type1^PVT^ neurons, time-locked to cue onset, sorted by latency to approach the reward zone, and binned into 5 ‘approach latency blocks’, L1, n = 61 trials, L2, n= 67 trials, L3, n = 67 trials, L4, n = 67 trials, L5, n = 64 trials from 6 mice. **(H)** *Left*: Latencies to reach the reward zone in seconds for each approach latency block. Repeated measures ANOVA, ****p<0.0001. Right: Velocity (m/s) during reward approach calculated for each approach latency block. Repeated measures ANOVA, ****p<0.0001. **(I)** Average GCaMP6s responses for fast and slow reward approach. Red line represents 20–80% of the slope-of-the-line. **(J)** Slope-of-the-line quantifications of GCaMP6s activity from Type1^PVT^ neurons across approach latency blocks. Repeated measures ANOVA, **p<0.01. **(K)** *Left*: Pairwise correlation between the approach latency and the slope-of-the-line quantifications of GCaMP6s responses from Type1^PVT^ neurons during approach. *Right*: Correlation between the velocity (m/s) during approach and the slope-of-the-line quantifications of GCaMP6s responses from Type1^PVT^ neurons during approach. Each dot represents the GCaMP6s activity of individual mice averaged across approach latency blocks. **(L)** Max peak quantifications of the reward approach-evoked changes in GCaMP6s activity across approach latency blocks. Repeated measures ANOVA, *p=0.19*; ns, not significant **(M)***. Left*: No correlation between approach latency and max peak. *Right*: No correlation between the max peak and the slope-of-the-line quantifications of GCaMP6s responses from Type1^PVT^ neurons during approach. **(N)** Average GCaMP6s fluorescence from Type1^PVT^ neurons during spontaneous locomotion. **(O)** Same as (G) but responses were sorted by trial order and binned into 5 ‘trial group blocks’. G1, n = 61 trials, G2, n= 67 trials, G3, n = 67 trials, G4, n = 67 trials, G5, n = 64 trials from 6 mice. Yellow dots represent reward zone arrival. **(P)** Latencies to reach the reward zone across trial group blocks. Repeated measures ANOVA, **p<0.001. **(Q)** No correlation between the approach latency and trial order. **(R)** Average GCaMP6s responses from Type1^PVT^ neurons comparing approach trials performed early and late in the testing session. Red line indicates 20–80% of the slope-of-the-line. **(S)** Max peak quantification of the reward approach-evoked changes in GCaMP6s activity across trial group blocks. Repeated measures ANOVA, **p<0.001. **(T)** Correlation between the max peak of the reward approach-evoked changes in GCaMP6s activity and trial order within a test session. **(U)** Slope-of-the-line quantifications of GCaMP6s activity across trial group blocks. Repeated measures ANOVA, *p=0.36*. **(V)** *Left*: Average GCaMP6s responses from Type1^PVT^ neurons upon reward delivery comparing early and late trials in the testing session. *Right*: Max peak quantification during reward delivery across trial group blocks. Repeated measures ANOVA, *p=0.34*; ns, not significant. **(W)** *Left*: Latencies to reach the reward zone across trial group blocks. Repeated measures ANOVA, *p=0.25*; ns, not significant. *Middle*: Average GFP activity in the PVT comparing approach trials performed early or late in the testing session. *Right*: Max peak quantification of GFP activity in the PVT during approach across trial group blocks. Repeated measures ANOVA*, p=0.78*; ns, not significant. All data in the figure are shown as mean ±s.e.m.

We measured the activity of Type1^PVT^ neurons while food-restricted mice performed trials in our foraging-like reward task. For this, we injected the PVT of *Drd2*-Cre mice with an AAV driving expression of a Cre-dependent calcium sensor (GCaMP6s) and recorded bulk fluorescence signals from this neuronal population using fiber photometry (See Methods; Fig. 1A). Following this approach, we found that Type1^PVT^ neurons were robustly activated as mice approached the reward zone and the receptacle to obtain the food reward (strawberry Ensure^®^; Fig. 1B). Modest but significant increases in calcium signals were also observed in these neuronal populations upon reward delivery (Fig. 1C). Importantly, no such changes in fluorescence were observed in control mice in which Type1^PVT^ neurons expressed GFP instead of GCaMP (Fig. 1D–F). Collectively, these results demonstrate that Type1^PVT^ neurons are modulated by reward-seeking and consumption.

To determine whether the activity of Type1^PVT^ neurons varied with the motivational state of the subjects, we ranked all trials based on the latency to reach the reward zone (Fig. 1G) and divided them into five categories (L1–L5; Supp. Fig. 2). Trials with the shortest latencies to the reward zone (i.e., trials performed within 2– 3 sec; Fig. 1H) were categorized as ‘fast trials’ (L1), whereas trials with the longest latencies (i.e., trials performed within 9–11 sec; Fig. 1H) were categorized as ‘slow trials’ (L5). We next compared the activity of Type1^PVT^ neurons during fast trials vs. slow trials. Our analyses revealed that during fast trials, Type1^PVT^ neurons appeared to display higher GCaMP signal ramps compared to slow trials (Fig. 1I). These differences in the GCaMP signal ramps were quantified by calculating the 20–80% slope of the peak of GCaMP transients observed during the reward approach. Quantifications of the signal’s slope confirmed significant differences in the activity of Type1^PVT^ neurons between fast and slow trials (Fig. 1J). Moreover, we found that both latency and velocity parameters were positively correlated with the GCaMP signals (Fig. 1K). Surprisingly, quantifications of the maximum peak of the signal (Fig.1L) revealed no significant differences between fast and slow trials. Accordingly, no association was found between the latency to complete trials and peak amplitude or between the approach peak and the approach slope (Fig. 1M). Lastly, no changes in the activity of Type1^PVT^ neurons were observed when mice were moving around the maze but were not engaged in the task (Fig. 1N). Altogether, our findings thus far suggest that Type1^PVT^ neurons are modulated by reward-seeking and consumption and that their activity varies with the motivational state of subjects.

To test if changes in need states impact Type1^PVT^ neuronal activity, we sorted trials by trial order, categorizing the earliest trials as ‘G1’ and the latest trials as ‘G5.’ (Fig. 1O – U). We implemented this strategy based on the assumption that well trained food-restricted mice are hungrier at the beginning compared to the end of each 60 min testing session^22^. Importantly, the latencies to approach the reward did not significantly differ across five trial groups distributed throughout testing sessions (Supp. Fig. 1M; Fig.1Q). Previous research has shown that NAc-projecting neurons of the posterior PVT (pPVT) are engaged by hunger signals and changes in glucose levels, suggesting that need states influence the activity of PVT neurons^10,13,23.^. In agreement with this view, we found that the activity of Type1^PVT^ neurons was higher during early trials than in later trials, indicating that Type1^PVT^ neuronal activity is modulated by the satiety levels of subjects (Fig. 1R–T). Of note, quantifications of the slope revealed no significant differences between early and late trials (Fig. 1U). We also found that the activity of Type1^PVT^ neurons at reward delivery trended towards a reduction as the session progressed, but these changes were not statistically significant (Fig. 1V). Importantly, the observed decreases in the activity of Type1^PVT^ neurons were unlikely due to photobleaching of the GCaMP signal, as no significant decreases in fluorescence signal were observed in GFP controls (Fig.1W). To probe our conclusions further, GCaMP-expressing mice were tested in a separate session where the reward was completely omitted for all trials. We observed that, under these conditions, Type1^PVT^ neurons were highly active across trials (Supp. Fig. 3A–F), and unlike for rewarded trials this activity did not vary throughout sessions (Supp. Fig. 3G–J). Notably, ‘fast trials’ still displayed significantly higher GCaMP ramps compared to ‘slow trials’, again supporting the notion that the activity dynamics of Type1^PVT^ neurons reflect behavioral vigor (Supp. Fig. 3K–N). Altogether, these findings suggest that Type1^PVT^ neuronal activity is influenced by satiety levels, with higher activity observed when mice are hungry and lower activity observed as satiety levels increase.

### Type2^PVT^ neuronal activity dynamics during food-seeking behavior

**Figure 2.**
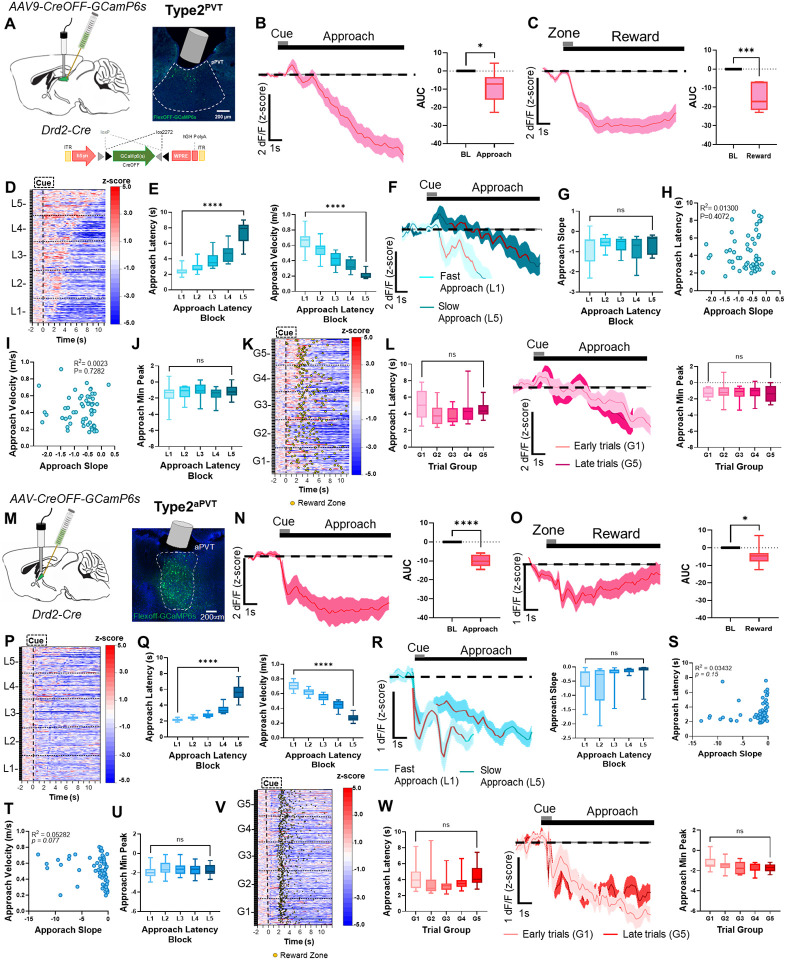
*In-vivo* activity dynamics of Type2^PVT^ neurons. **(A)** *Left*: Schematic of the stereotaxic injections for selective expression of Cre-OFF GCaMP6s viral vectors, as well as fiber implantation to target Type2^PVT^ neurons in the pPVT. *Right*: Representative image from a Drd2-Cre mouse expressing Cre-OFF GCaMP6s in the pPVT. **(B)** *Left*: Average GCaMP6s response from Type2^PVT^ neurons during reward approach. *Right*: Quantification of the approach-evoked changes in GCaMP6s fluorescence in Type2^PVT^ neurons. AUC, n = 420 trials from 6 mice, two-tailed paired t-test, *p<0.05. **(C)** *Left*: Average GCaMP6s responses from Type2^PVT^ neurons during reward delivery. *Right*: AUC quantification of reward-evoked changes in GCaMP6s fluorescence in Type2^PVT^ neurons. Two-tailed paired t-test, ***p<0.001. **(D)** Heatmap showing approach responses from Type2^PVT^ neurons, time-locked to cue onset, sorted by latency to approach the reward zone, and binned into 5 ‘approach latency blocks’, L1, n = 79 trials, L2, n= 81 trials, L3, n = 84 trials, L4, n = 87 trials, L5, n = 89 trials from 6 mice. **(E)** *Left*: Latencies to reach the reward zone for each latency block. Repeated measures ANOVA ****p<0.0001. *Right*: Velocity (m/s) during reward approach calculated for each latency block. Repeated measures ANOVA, ****p<0.0001. **(F)** Average GCaMP6s dynamics for fast approach (L1) and slow approach (L5) in Type2^PVT^ neurons. Red line indicates 20–80% of the slope-of-the-line. **(G)** Slope-of-the-line quantifications of GCaMP6s activity from Type2^PVT^ neurons across approach latency blocks. Repeated measures ANOVA, *p=0.25*; ns, not significant. **(H)** Correlation between the approach latency and the slope-of-the-line quantifications of GCaMP6s responses during reward approach. Each dot represents the GCaMP6s activity of individual mice averaged across approach latency blocks. **(I)** Correlation between the velocity (m/s) during approach and the slope-of-the-line quantifications of GCaMP6s responses during reward approach. **(J)** Min peak quantification of the reward approach-evoked changes in GCaMP6s activity from Type2^PVT^ neurons across approach latency blocks. Repeated measures ANOVA, *p=0.78*; ns, not significant. **(K)** Same as (D) but responses were sorted by trial order and binned into 5 ‘trial group blocks,’ G1, n = 79 trials, G2, n= 81 trials, G3, n = 84 trials, G4, n = 87 trials, G5, n = 89 trials from 6 mice. Yellow dots represent reward zone arrival. **(L)** *Left*: Latencies to reach the reward zone across trial group blocks. Repeated measures ANOVA, *p=0.11*; ns, not significant*. Middle*: Average GCaMP6s responses from Type2^PVT^ neurons comparing approach trials performed early and late in the testing session. *Right*: Min peak quantification of the reward approach-evoked changes in GCaMP6s activity from Type2^PVT^ neurons across trial group blocks. Repeated measures ANOVA, *p=0.95*; ns, not significant. **(M)** *Left*: Schematic of stereotaxic injections and fiber implantation. *Right*: Representative image of Type2^aPVT^ neurons in the aPVT. **(N)** *Left*: Average GCaMP6s response from Type2^aPVT^ neurons showing decreases in activity dynamics during reward approach. *Right*: AUC quantification of approach-evoked changes in GCaMP6s fluorescence in Type2^aPVT^ neurons. AUC, n = 650 trials from 8 mice, two-tailed paired t-test, ****p<0.0001. **(O)** *Left*: Average GCaMP6s response from Type2^aPVT^ neurons showing decrease in activity dynamics during reward delivery*. Right*: AUC quantification of reward-evoked changes in GCaMP6s fluorescence in Type2^aPVT^ neurons. Two-tailed paired t-test, *p<0.05. **(P)** Heatmap showing approach responses of Type2^aPVT^ neurons, time-locked to cue onset, sorted by latency to approach the reward zone, and binned into 5 ‘approach latency blocks’ (L1 – L5) , L1, n = 129 trials, L2, n= 129 trials, L3, n = 129 trials, L4, n = 129 trials, L5, n = 139 trials from 8 mice. **(Q)** *Left*: Latencies to reach the reward zone in seconds (s) for each approach latency block. Repeated measures ANOVA, ****p<0.0001. *Right*: Velocity (m/s) during reward approach calculated for each approach latency block. Repeated measures ANOVA, ****p<0.0001. **(R)** *Left*: Average GCaMP6s responses for fast and slow reward approach. Red line indicates 20–80% of the slope-of-the-line. *Right*: Slope-of-the-line quantifications of GCaMP6s activity from Type2^aPVT^ neurons across approach latency blocks. Repeated measures ANOVA, *p=0.08*; ns, not significant **(S)** Correlation between approach latency and the slope-of-the-line quantifications per animal for each latency block. **(T)** Correlation between approach velocity and the slope-of-the-line of the line quantifications **(U)** Min peak quantification during reward approach across latency blocks. Repeated measures ANOVA, *p=0.22*; ns, not significant. **(V)** Same as (P) but responses were sorted by trial order and binned into 5 ‘trial group blocks’ (G1 – G5). Yellow dots represent reward zone arrival. **(W)** *Left*: Quantification of latency of reward approach across trial group blocks. Repeated measures ANOVA, *p<0.05; G1 vs. G5 Tukey’s multiple comparisons test, *p=0.99*. *Middle*: Average GCaMP6s response for Type2^aPVT^ neurons during trials performed early (G1) and late (G5) during the session. *Right*: Min peak quantification of GCaMP6s activity during approach across trial group blocks. Repeated measures ANOVA, *p=0.10*; ns, not significant. All data in the figure are shown as mean ±s.e.m.

We next examined the Type2^PVT^ neuronal population and asked whether their activity dynamics during the reward foraging task differ from that of Type1^PVT^ neurons. For this, the pPVT of *Drd2*-Cre mice was injected with an AAV driving expression of GCaMP6s exclusively in Cre negative neurons (CreOFF; Type2^pPVT^) and used fiber photometry to measure their activity while well-trained mice performed the task (Fig. 2A). We found that, unlike Type1^PVT^ neurons, the activity of Type2^pPVT^ neurons significantly decreased during both reward approach and reward delivery (Fig. 2B, C). Given that, like Type1^PVT^ neurons, the activity of Type2^PVT^ neurons is also modulated during reward approach and delivery, we then sought to assess whether the activity of Type2^PVT^ neurons could also be influenced by motivational factors. To group the trials based on their latency to reach the reward zone (Fig. 2D), we sorted them again and classified into five blocks of increasing latency from L1 (lowest) to L5 (highest) (Supp. Fig. 2C; Fig. 2E).

Interestingly, the slope of the ramps did not significantly differ between the two trial types (Fig. 2F, G), nor was there any correlation between the slope and the latency to reward approach or trial velocity (Fig. 2H, I). There was also no significant effect on minimum peak amplitudes for fast and slow trials (Fig. 2J). These findings indicate that Type2^PVT^ neuronal activity decreases during the reward approach, but this decrease is not influenced by the degree of behavior vigor. Previous studies have also suggested that the anterior PVT (aPVT), where Type2^PVT^ neurons are more abundant^17^, modulates food-seeking and feeding behavior and is innervated by hypothalamic neurons that signal satiety^7,33^. Therefore, we investigated whether the activity of Type2^PVT^ neurons varied based on the satiety levels of the mice. To this end, we compared the activity of Type2^PVT^ neurons during the early (G1) and late (G5) trials of testing sessions (Fig. 2K, L). However, our analysis revealed no significant differences in the GCaMP signals of Type2^PVT^ neurons between early and late trials (Fig. 2L). Of note, significant differences in latency were not observed for G1 and G5 in this cohort. Lastly, similar dynamics were observed in the aPVT (Fig. 2M–W). Overall, our findings suggest that unlike Type1^PVT^ neurons, changes in Type2^PVT^ neuronal activity during the reward approach are not modulated by the degree of motivation or satiety levels of the animals.

### Type1^PVT^ and Type2^PVT^ neurons activity dynamics during trial termination

**Figure 3.**
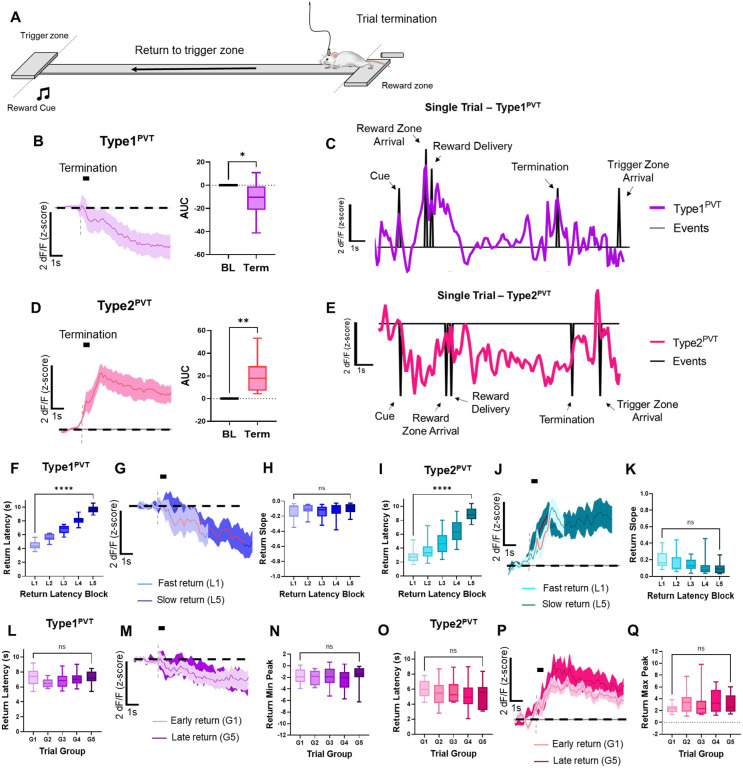
Type1^PVT^ and Type2^PVT^ neurons *in vivo* dynamics during trial termination. **(A)** Schematic depicting animal return to the trigger zone in our foraging-like reward seeking task. **(B)** *Left*: Average GCaMP6s responses from Type1^PVT^ neurons during trial termination and return. *Right*: Quantification of the return-evoked changes in GCaMP6s fluorescence in Type1^PVT^ neurons. AUC, two-tailed paired t-test, *p<0.05. **(C)** In a complete trial, the activity dynamics of Type1^PVT^ neurons from a sample subject during the reward foraging task are represented by the purple line, while the black ticks indicate various events within the trial. **(D)** *Left*: Average GCaMP6s responses from Type2^PVT^ neurons during trial termination and return. *Right*: Quantification of the return-evoked changes in GCaMP6s fluorescence in Type2^PVT^ neurons. AUC, two-tailed paired t-test, **p<0.01. **(E)** Same as (C) for Type2^PVT^ neurons. **(F)** Latencies to reach the trigger zone across approach latency blocks for Type1^PVT^ neuronal imaging. Repeated measures ANOVA, ****p<0.01. **(G)** Average GCaMP6s dynamics for fast return (L1) and slow return (L5) in Type1^PVT^ neurons. Red line indicates 20–80% of the slope-of-the-line. **(H)** Slope-of-the-line quantification of GCaMP6s return activity in Type1^PVT^ neurons. Repeated measures ANOVA, *p=0.45*; ns, not significant. **(I)** Latencies to reach the trigger zone across approach latency blocks for Type2^PVT^ photometry recordings. Repeated measures ANOVA, ****p<0.01 **(J)** Average GCaMP6s dynamics for fast return (L1) and slow return (L5) in Type2^PVT^ neurons. Red line indicates 20–80% of the slope-of-the-line. **(K)** Slope-of-the-line quantification of GCaMP6s return activity in Type2^PVT^ neurons across latency blocks. Repeated measures ANOVA, *p=0.28*; ns, not significant. **(L)** Latencies to reach the trigger zone across trial group blocks for Type1^PVT^ photometry recordings. Repeated measures ANOVA, *p=0.29*; ns, not significant. **(M)** Average GCaMP6s return dynamics from Type1^PVT^ neurons comparing return trials performed early and late in the testing session. **(N)** Min peak quantification of GCaMP6s activity during return in Type1^PVT^ neurons across trial group blocks. Repeated measures ANOVA, *p=0.47*; ns, not significant. **(O)** Latencies to reach the trigger zone across trial group blocks for Type2^PVT^ photometry recordings. Repeated measures ANOVA, *p=0.18*; ns, not significant. **(P)** Average GCaMP6s return dynamics in Type2^PVT^ neurons for early and late trials. **(Q)** Max peak quantification of GCaMP6s activity during return in Type2^PVT^ neurons across trial group blocks. Repeated measures ANOVA, *p=0.25*; ns, not significant. All data in the figure are shown as mean ±s.e.m.

Previous studies, including those from our own group, have established that PVT neuronal activation is crucial for food-seeking behavior^13,23–26^. We now provide evidence that Type1^PVT^ neurons are likely implicated in this process. However, other studies have suggested that PVT neuron activation might also promote the suppression of reward-seeking behavior^7,27,28^. This led us to hypothesize that Type2^PVT^ neurons, albeit being suppressed during the initiation of goal pursuits, signal the termination of such behaviors. To test this prediction, we examined the *in vivo* activity dynamics of both Type1^PVT^ and Type2^PVT^ neurons during ‘trial termination,’ defined as the moment when mice completed reward consumption and began to return to the trigger zone to initiate another trial (Fig. 3A). Our analysis revealed that trial termination resulted in a decrease in Type1^PVT^ neuron activity (Fig. 3B, C) and significant increases in Type2^PVT^ neuron activity (Fig. 3D, E), supporting our hypothesis that Type2^PVT^ neurons signal the termination of food seeking behavior. Interestingly, when trials were sorted and binned by latency to return to the trigger zone, neither Type1^PVT^ nor Type2^PVT^ neuronal activity was modulated by whether the mice performed a ‘slow’ or ‘fast’ return (Fig. 3F–K). Additionally, when trials were sorted and binned by trial order, we found no significant modulation by the hunger state of the animals for either neuronal type (Fig. 3L–Q). In conclusion, our findings indicate that Type2^PVT^ neuronal activity, but not Type1^PVT^, signals the termination of goal pursuits.

### Type1^PVT^–NAc and Type2^PVT^–NAc terminals display similar activity dynamics to Type1^PVT^ and Type2^PVT^ neurons.

**Figure 4.**
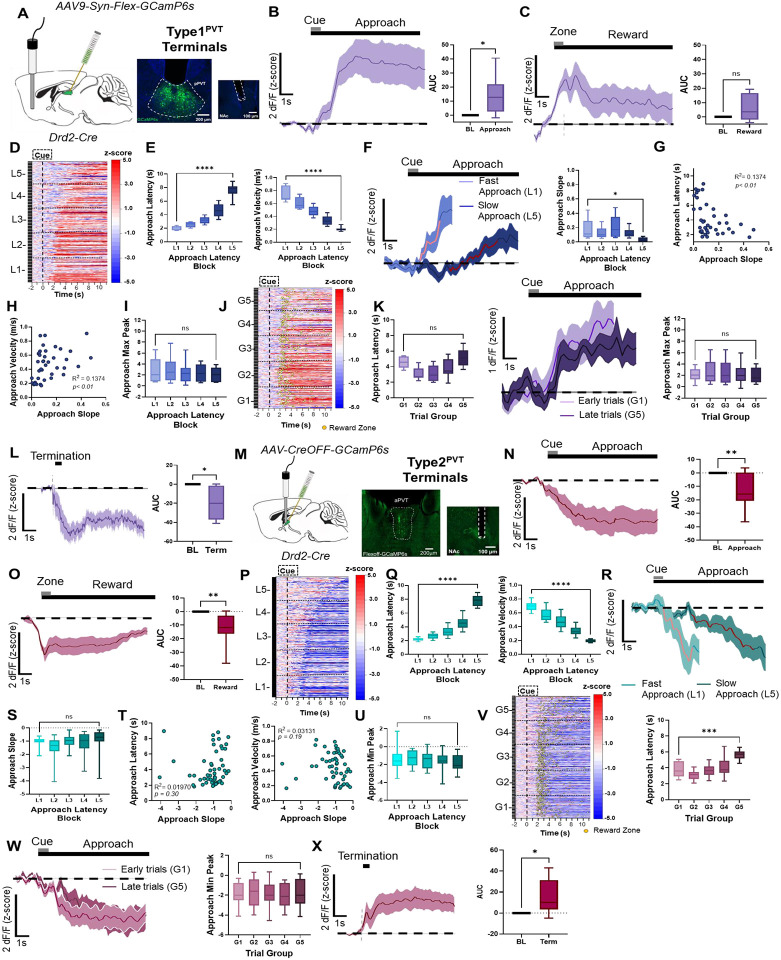
*In vivo* activity dynamics of Type1^PVT^–NAc and Type2^PVT^–NAc axon terminals. **(A)** *Left*: Schematic of stereotaxic injections and fiber implantation. *Right*: Representative images of GCaMP6s expression in Type1^PVT^ neurons in the pPVT and fiber placement in NAc. **(B)** *Left*: Average GCaMP6s approach-evoked responses from Type1^PVT^ terminals during reward approach. *Right*: Quantification of the approach-evoked changes in GCaMP6s fluorescence in Type1^PVT^ terminals. AUC, n = 330 trials from 4 mice, two-tailed paired t-test, *p<0.05. **(C)** *Left*: GCaMP6s reward-evoked response in Type1^PVT^ terminals during reward delivery. *Right*: Quantification of the reward-evoked changes in GCaMP6s fluorescence in Type1^PVT^ terminals. AUC, two-tailed paired, *p=0.08*; ns, not significant. **(D)** Heatmap showing excitatory reward approach responses of Type1^PVT^ terminals, time-locked to cue onset, sorted by latency to approach the reward zone, and binned into 5 ‘approach latency blocks’ (L1 – L5). L1, n = 65 trials, L2, n= 65 trials, L3, n = 65 trials, L4, n = 65 trials, L5, n = 70 trials from 4 mice. **(E)** *Left*: Latency to approach reward in seconds for Type1^PVT^ terminals across latency blocks. Repeated measures ANOVA, ****p<0.0001. Right: Velocity (m/s) during reward approach calculated across each latency block. Repeated measures ANOVA, ****p<0.0001. **(F)** *Left*: Average GCaMP6s Type1^PVT^ terminal dynamics for fast approach (L1) and slow approach (L5). Red line indicates 20–80% of the slope-of-the-line. *Right*: Slope-of-the-line quantifications of GCaMP6s activity from Type1^PVT^ terminals across approach latency blocks. Repeated measures ANOVA, *p<0.05. **(G)** Correlation between the approach latency and the slope-of-the-line quantifications of GCaMP6s responses during reward approach in Type1^PVT^ terminals. **(H)** Correlation between the approach velocity and the slope-of-the-line during reward approach. **(I)** Max peak quantification of the reward approach-evoked changes in GCaMP6s activity across approach latency blocks. Repeated measures ANOVA, *p=0.40*; ns, not significant. **(J)** Same as (D) but sorted by trial order and binned into 5 ‘trial group blocks’ (G1-G5). Yellow dots represent reward zone arrival. **(K)** *Left*: Latency to approach reward in seconds for Type1^PVT^ terminals across latency blocks. Repeated measures ANOVA, ****p<0.0001. Middle: Average GCaMP6s approach dynamics of Type1^PVT^ terminals comparing approach trials performed early and late in the session. *Right*: Max peak quantification of the approach-evoked changes in GCaMP6s activity from Type1^PVT^ terminals across trial group blocks. Repeated measures ANOVA, *p=0.51*; ns, not significant. **(L)** *Left*: Average GCaMP6s responses from Type1^PVT^ terminals during trial termination and return. *Right*: Quantification of the return-evoked changes in GCaMP6s fluorescence in Type1^PVT^ terminals. AUC, two-tailed paired t-test, *p<0.05. **(M)** *Left*: Schematic of stereotaxic injections and fiber implantation. *Right*: Representative images of GCaMP6s expression in Type2^PVT^ neurons in the aPVT and fiber placement in NAc. **(N)** *Left*: Average GCaMP6s approach-evoked responses from Type2^PVT^ terminals during reward approach. *Right*: Quantification of the approach-evoked changes in GCaMP6s in Type2^PVT^ terminals. AUC, n = 646 trials from 6 mice, two-tailed paired t-test, **p<0.01. **(O)** *Left*: Average GCaMP6s responses from Type2^PVT^ terminals during reward delivery. *Right*: Quantification of reward-evoked changes in GCaMP6s. AUC, two-tailed paired t-test, **p<0.01. **(P)** Heatmap showing inhibitory approach responses from Type2^PVT^ terminals, time-locked to cue onset, sorted by latency to approach the reward zone, and binned into 5 ‘approach latency blocks’ (L1 – L5). L1, n = 127 trials, L2, n= 129 trials, L3, n = 127 trials, L4, n = 130 trials, L5, n = 133 trials from 6 mice. **(Q)** *Left*: Latencies to reach the reward zone in seconds for each approach latency block. Repeated measures ANOVA, ****p = 0.0001. *Right*: Velocity (m/s) during reward approach calculated for each approach latency block. Repeated measures ANOVA, ****p<0.0001. **(R)** Average GCaMP6s dynamics for Type2^PVT^ terminals during fast and slow trials. **(S)** Slope-of-the-line quantifications of GCaMP6s activity from Type2^PVT^ terminals across approach latency blocks. Repeated measures ANOVA, *p=0.42*; ns, not significant. **(T)** *Left*: No correlation between the approach latency and the slope-of-the-line quantifications of GCaMP6s responses from Type2^PVT^ terminals during approach. *Right*: No correlation between approach velocity and the slope-of-the-line quantifications of GCaMP6s responses during reward approach. **(U)** Min peak quantification of the approach-evoked changes in GCaMP6s activity across approach latency blocks. Repeated measures ANOVA, *p=0.35*; ns, not significant. ns, not significant. **(V)** *Left*: Same as (P), but responses were sorted by trial order and binned into 5 ‘trial group blocks’ (G1 – G5). Yellow dots represent reward zone arrival. *Right*: Latencies to reach reward across trial group blocks for Type2^PVT^ terminals. Repeated measures ANOVA, ***p<0.001. **(W)** *Left*: Average GCaMP6s Type2^PVT^ terminal dynamics comparing trials performed early and late in the session. *Right*: Min peak quantification of the approach-evoked changes in GCaMP6s activity from Type2^PVT^ terminals across trial group blocks. Repeated measures ANOVA, *p=0.51*; ns, not significant. **(X)** *Left*: Average GCaMP6s responses from Type2^PVT^ terminals during trial termination and return. *Right*: Quantification of the return-evoked changes in GCaMP6s fluorescence in Type2^PVT^ terminals. AUC, two-tailed paired t-test, *p<0.05. All data in the figure are shown as mean ±s.e.m.

Type1^PVT^ and Type2^PVT^ neurons project to the NAc, a brain region that plays a critical role in the learning and execution of goal-oriented behaviors^29–32^. For this reason, to investigate how Type1^PVT^ and Type2^PVT^ neurons contribute to goal-oriented behaviors via their projections to the NAc, we assessed the activity dynamics of Type1^PVT^–NAc and Type2^PVT^–NAc terminals at different stages of our reward foraging behavioral task (Fig. 4). We used the same clustering analyses to compare the activity dynamics of these terminals between trials with different latencies and those at different stages of the session (Supp. Fig. 3E, F). Our results demonstrate that Type1^PVT^–NAc terminals mirror the activity dynamics of Type1^PVT^ neurons (Fig. 4A – L). Specifically, we observed robust increases in the fluorescence of Type1^PVT^–NAc terminals during the reward approach, followed by a bi-phasic response at reward delivery (Fig. 4A – C). In addition, Type1^PVT^–NAc terminals showed higher GCaMP signal ramps during fast trials compared to slow trials (Fig. 4D –F). We also found a negative correlation between approach latency and the slope of GCaMP signals, as well as a positive correlation between velocity to approach the reward and the slope of the GCaMP signal (Fig. 4G, H). Surprisingly, we observed no significant differences in the GCaMP signals of Type1^PVT^–NAc terminals between early and late trials (Fig. 4J – K). We also found that Type1^PVT^–NAc terminals showed a significant decrease in activity during trial termination (Fig. 4L). Finally, analysis of Type2^PVT^–NAc terminal activity revealed similar dynamics to the broader Type2^PVT^ neuronal population (Fig. 4M – X). Type2^PVT^–NAc terminals displayed significant decreases in activity during the reward approach and delivery (Fig. 4N, O). However, analysis of trial type showed no significant differences in Type2^PVT^–NAc terminals’ activity between fast vs. slow approach or early vs. late trials (Fig.4P –W). Lastly, trial termination resulted in increases in the activity of Type2^PVT^–NAc terminals (Fig. 4X). Our findings strongly suggest that motivation-related features and the encoding of goal-oriented actions of Type1^PVT^ and Type2^PVT^ neurons are being relayed to the NAc through their respective terminals.

## DISCUSSION

We have identified two thalamo-striatal pathways, Type1^PVT^–NAc and Type2^PVT^–NAc, whose *in vivo* dynamics respectively encode the initiation and termination of goal pursuits. In addition, we found that one of these two pathways, the Type1^PVT^–NAc, selectively tracks the motivational state of subjects. These results offer a framework for interpreting seemingly contradictory findings from the literature, where manipulations of the PVT either increase or decrease reward seeking^7,10,13,23,25,26,33^. Indeed, our studies support the notion that the dorsal midline thalamus integrates signals about need states to shape goal-oriented behaviors^9,14,20,24,34,35^ .

The Type1^PVT^–NAc and Type2^PVT^–NAc pathways appear to serve complementary functions in the initiation and termination of goal-oriented behaviors. Interestingly, their activity dynamics appear to be largely mutually exclusive, with Type1^PVT^ neurons activated at initiation when Type2^PVT^ activity is suppressed, and Type2^PVT^ neurons becoming activated at termination when Type1^PVT^ activity is reduced. While this pattern of activity suggests an inhibitory relationship exists between these two neuronal classes, the rodent thalamus (including PVT) is largely devoid of interneurons^36^, and Type1^PVT^ and Type2^PVT^ neurons are not synaptically connected to one another^17,37^. One potential mechanism for mediating the inhibitory interaction between these two functional subclasses of the PVT is the thalamic reticular nucleus (TRN). The TRN is a thin sheet of GABAergic neurons that surrounds the thalamus and is known to play a key role in gating sensory information by modulating thalamo-cortical transmission^38^. Recent work confirms earlier anatomical models suggesting that the TRN mediates interactions between different thalamic nuclei^39–42^. In the context of the PVT, it is possible that the TRN plays a similar role in mediating interactions between the Type1^PVT^ and Type2^PVT^ pathways. Specifically, the TRN may act as a hub that receives input from both pathways and modulates their activity through inhibitory interactions, resembling an open-loop configuration^43,44^. Of note, recent experimental evidence supports the existence of an open-loop configuration involving PVT and TRN^45^. Such architecture could allow for fine-tuning of goal-oriented behavior by ensuring that the appropriate PVT–NAc pathway is activated or inhibited at any given time. While further work is needed to test this hypothesis, our results suggest that the TRN may be a key player in the neural circuits of motivated behaviors.

Our findings that Type1^PVT^–NAc neurons, and in particular their projections to the NAc encodes the vigor or intensity of goal-directed behavior are consistent with previous studies linking dopamine (DA) dynamics in the NAc to behavioral activation^46^. Considering the well-established role of the PVT in arousal^47–49^ , a necessary component that enables behavioral activation^50,51^, Type1^PVT^ neurons may play a critical role in promoting need-based arousal states that are translated into motivational signals within the NAc^48,52^. Accordingly, NAc neurons have been reported to encode motivational parameters such as the initiation and vigor of reward seeking^52–54^. Intriguingly, the ramping in neuronal activity observed in Type1^PVT^ neurons resembled those observed when measuring dopamine signaling from the mesoaccumbens pathway during motivated behaviors^54–56^. Similarly, the slope of the ramp for dopaminergic signaling has been shown to be modulated by speed, such that fast reward approach results in steeper slopes than slow reward approach^55^. Furthermore, the ramping of dopaminergic signaling significantly decreases when a small, or no reward, is delivered^55,56^. However, unlike dopamine ramps, reward value does not seem to influence the slope of the Type1^PVT^ neuronal activity ramps, as shown by the maintenance of the ramping Type1^PVT^ neuronal activity during the reward omission session. Instead, the magnitude and peaks responses of the Type1^PVT^ activity seem to encode motivational states such as hunger. In agreement with these findings, recent studies have provided insights into the neural circuits underlying the PVT’s role in processing internal states. Specifically, the hypothalamus and brainstem have been identified as key players in transmitting information about an organism’s internal state to the PVT^10–13,57^. For instance, a previous study^13^ demonstrated that orexinergic neurons projecting to the PVT regulate feeding behavior, highlighting the role of hypothalamic signaling in controlling the motivational value of food. Another recent study^12^ showed that hypothalamic projections to the PVT drive food odorant attraction in hungry mice, providing further evidence for the role of hypothalamic input in mediating goal-directed behavior. These findings support the notion that need-based signals are conveyed to the PVT to regulate motivated behaviors in response to changes in internal states. Here, we have suggested that Type1^PVT^ neurons projecting to the NAc might be mediating this process.

Over the past decade, there has been a growing body of work exploring the role of the PVT in motivated behaviors^9,13,20,23–26,52^. However, the precise contribution of the PVT to these behaviors has remained unclear. Here we have shown that two major subtypes of PVT neurons differentially signal the initiation and termination of reward seeking, likely through their projections to the NAc. It is important to note that we classified PVT neurons as Type1^PVT^ and Type2^PVT^ based on their expression or lack thereof the dopamine D2 receptor, respectively, and as such it is possible that other subpopulations of PVT neurons may also contribute to goal-oriented behavior. In fact, recent research using single-cell RNA sequencing has identified at least 5 potential molecularly distinct PVT subtypes^16^. As such, Type1^PVT^ neurons can be further subdivided into two subtypes: *Esr1*^+^ and *Col12a1*^+^. While Type2^PVT^ can also be subdivided into two subtypes: *Npffr1*^+^, *Hcrtr1*^+^. A fifth subpopulation of PVT neurons is distributed across the antero-posterior axis of the PVT and expresses *Drd3*^16^. Thus, while our findings provide valuable insights into the role of PVT subtypes in motivated behaviors, future studies should investigate the contributions of other PVT subpopulations to fully understand the complex dynamics of the PVT–NAc circuitry.

The findings presented here shed light on the neural circuits that underlie motivated behaviors, including initiation, vigor, and termination. These processes are central to achieving goals and maintaining appropriate levels of motivation in everyday life. However, deficits in motivation are associated with a range of psychiatric conditions, such as substance abuse, binge eating, and anhedonia in depression^29,58–60^. Therefore, a deeper understanding of the neural basis of motivated behavior may offer new insights into the treatment of these debilitating conditions. By revealing the specific neuronal pathways involved in motivation and how they interact, this research may provide new therapeutic targets for interventions aimed at restoring healthy motivational processes in individuals with such conditions.

## METHODS

### Mice

For all experiments, both male and female *Drd2*-Cre mice (8–15 weeks of age) were used. These mice were obtained from the GENSAT (founder line ER44) and were group housed under a 12-h light-dark cycle (6 a.m. to 6 p.m. light), at a temperature of 70–74 °F and 40–65% humidity. After surgery, mice were single-housed and were provided with food and water *ad-libitum*. Two days before initiating behavioral training procedures, food was restricted, and mice were fed accordingly to maintain 85% of their free-feeding weight. After all testing was completed, mice returned to *ad-libitum* feeding. Animals were randomly allocated to the different experimental conditions reported in this study. Importantly, all procedures were performed in accordance with the *Guide for the Care and Use of Laboratory Animals* and were approved by the National Institute of Mental Health (NIMH) Animal Care and Use Committee.

### Viral vectors

AAV9-hSyn-FLEX-GCaMP6s-WPRE-SV40 was produced by the Vector Core of the University of Pennsylvania. AAV9-CAG-Flex-GFP was produced by the University of North Carolina, Chapel Hill (UNC) Vector Core. AAV9-Syn-DO-GCaMP6s was produced by Charu Ramakrishnan (Deisseroth laboratory, Stanford University, CA, USA).

### Stereotaxic surgery

Mice were first anesthetized with a Ketamine/Xylazine solution, and an AngleTwo stereotaxic device (Leica Biosystems) was used for viral injections (approximately 1 μl) at the following stereotaxic coordinates (at a 6°angle): pPVT, −1.60 mm from Bregma, 0.06 mm lateral from midline, and −3.30 mm vertical from the cortical surface; aPVT, −0.30 mm from bregma, 0.00 mm lateral from the midline and −4.30 mm vertical from the cortical surface. For the fiber photometry experiments, optical fibers with diameters of 400 μm (0.66 NA - Doric Lenses) were used. These fibers were implanted over the pPVT or aPVT immediately following viral injections (targeted 300–400 μm above the injection site). NAc fibers coordinates were: 1.40 mm from bregma, 0.70 mm lateral from the midline, and −4.80 mm vertical from the cortical surface. Fibers were cemented using C&B Metabond Quick Adhesive Cement System (Parkell, Inc.) and Jet Brand dental acrylic (Lang Dental Manufacturing Co., Inc.). For analgesia and anti-inflammatory purposes postoperatively, mice received subcutaneous injections with metacam (meloxicam, 1–2 mg/kg) and were allowed to recover on a heating pad, where they were constantly monitored. Following stereotaxic injections, AAVs were allowed for 2–3 weeks for maximal expression.

### Foraging-like reward-seeking task

The foraging track consisted of a linear maze (150 × 32 × 25, L ×W× H in cm) containing digital distance sensors (15 cm, Pololu Robotics, and Electronics) located throughout the track, which allowed us to track the movement of the mice through the maze. The maze consisted of three zones: trigger zone, corridor, and reward zone. The opposite ends of the track were designated as the trigger zone and reward zone and were connected by a long corridor. Training in the task consisted of two initial sessions of magazine training. In which ¾ of the maze was closed, and mice received 100 μls of the reward every minute for 60 mins. Thereafter, mice were trained in the task for at least 14 days until they reliably performed at least 50 trials or more. The task consisted of 60 min-long sessions of self-paced trials and mice received one testing session per day. For each trial, food-restricted mice were trained to wait in the trigger zone for two seconds. An auditory cue was then presented, which signaled reward availability, allowing mice to run from the trigger zone down the corridor and into the reward zone to retrieve a food reward (strawberry Ensure^®^). Delivery and food consumption signaled trial termination, and therefore mice had to return to the trigger zone to initiate another trial. Experimental schedule and data acquisition were implemented through the Abet II software for operant control (Lafayette Instruments Neuroscience) and through the Whisker multimedia software (Lafayette Instruments Neuroscience).

### Bulk Ca^2+^ and fiber photometry

All photometry experiments were performed using an RZ5P acquisition system (Tucker-Davis Technologies; TDT) equipped with a real-time signal processor and controlled by a software interface (Synapse version 92). Specifically, the system is integrated with two continuous sinusoidally modulated LEDs (DC4100, ThorLabs) at 473 nm (211 Hz) and 405 nm (531 Hz) that serves as the light source to excite GCaMP6s and an isosbestic autofluorescence signal, respectively. The LED intensity (ranging 10–15 μW) at the interface between the fiber tip and the animal was constant throughout the session. Fluorescence signals were collected by the same fiber implant that was coupled to a 400 μm optical patch-cord (0.48 NA) and focused onto two separate photoreceivers (2151, Newport Corporation) connected to the emission ports of a custom-ordered fiber photometry Mini Cube (Doric Lenses). TTL pulses recorded by the same system were used to annotate the occurrence of behavioral manipulations. For the measurements of fluorescent calcium signals and ΔF/F analysis, a least-squares linear fit to the 405 nm signal to align it to the 470 nm signal was first applied. The resulting fitted 405 nm signal was then used to normalize the 473 nm as follows: ΔF/F = (473 nm signal − fitted 405 nm signal)/fitted 405 nm signal. All GCaMP6s signal data is presented as the z-score of the dF/F from a baseline prior to the onset of events.

### In-vivo dynamics analyses

Upon calculating the z-score of the dF/F for each event in every trial the mice performed. The latency to the reward zone was calculated. All trials that were higher than 11 seconds and the events corresponding to the trial (cue, approach, reward zone arrival, reward delivery, and consumption) were excluded. Approximately more than 75% of the trials met this inclusion criterion (Supp. Fig. 1L). After that, the trials and events for each individual testing session were sorted by either their latency to approach the reward zone (Approach Latency Blocks) or by trial order - the time in which the trial was completed within a testing session (Trial Group Blocks). After sorting the trials in each testing session, these were then divided into 5 equivalent trial blocks.

Slope-of-the-line calculations: Activity traces for reward approach, and reward termination included the activity from time 0, which corresponded to cue delivery, to 11 sec, which was our cut-off for included trials. The maximum and minimum peaks were calculated for each trace, and 80% and 20% values of the max or min peaks were determined. Thereafter, the slope-of-the-line was fitted for the corresponding 80% and 20% values.

### Histology

To verify GCaMP6s expression and optical fiber placements, mice were injected with euthanasia solution and subsequently sacrificed via transcardial perfusion, first with PBS and then with paraformaldehyde (PFA; 4% in PBS). Brains were then post-fixed in 4% PFA at 4 °C overnight and cryoprotected using a 30% PBS-buffered sucrose solution for ~24–36 h. Coronal brain sections (45 μm) were generated using a freezing microtome (SM 2010R, Leica). Images were taken using a Carl Zeiss LSM 780 confocal microscope running ZEN software (version 2.3, Carl Zeiss Microscopy, LLC). Optical fiber placements for all subjects included in this study are presented in Supplemental Fig. 4. Mice without correct targeting of optical fibers or GCaMP6s expression were excluded from this study.

### Statistics and data presentation

All data were analyzed using GraphPad Prism (Domatics) and Origin Pro 2016 (OriginLab Corp). All statistical tests are indicated when used. No assumptions or corrections were made prior to data analysis. For statistical analyses, a two-tailed paired t-test (nonparametric; test statistic: t) and a repeated measures ANOVA (nonparametric; test statistic: F) were used. ANOVA was followed by a post-hoc Tukey multiple comparisons test if omnibus test detected a significant difference. All data are presented as mean ± s.e.m. The sample sizes used in our study are similar to those of prior studies^10^. For all the groups tested, the sample size was at least 6 mice, with the exception of the terminal imaging of Type1^PVT^ neurons, for which there were 4 mice. (Supp. Fig. 2). This was due to technical challenges associated with performing calcium imaging from terminals in our task. All experiments were replicated at least once. The data were assumed to be normal, but this was not formally tested.

## Supplementary Material

1

## Data Availability

All the data that support the findings presented in this study are available from the corresponding author upon reasonable request.
