# Supplementary material for: Dissociable encoding of motivated behavior by parallel thalamo-striatal projections
Source: bioRxiv. 2024 Jan 21:2023.07.07.548113. Originally published 2023 Jul 7. Preprint. [Version 2] doi: 10.1101/2023.07.07.548113 (PMC10541145; doi:10.1101/2023.07.07.548113)

bioRxiv preprint doi: https://doi.org/10.1101/2023.07.07.548113; this version posted January 21, 2024. The copyright holder for this preprint (which was not certified by peer review) is the author/funder. This article is a US Government work. It is not subject to copyright under 17 USC 105 and is also made available for use under a CC0 license.

Supplemental Figure 1. Mice performance in foraging–like task to characterize motivated behavior in rodents and trial distribution per experimental group.

(A). Schematics depicting our foraging-like reward-seeking task.

(B) Quantification of trials completed during the first days of training (Days 1-2) and the last days of training (Days 13-14) for all the mice included in the study. Two-tailed paired t-test, ****$p<0.0001$.

(C) *Left:* Comparisons of the latencies to the reward zone. Lighter colors indicate the first days of training, and darker colors indicate the last days of training. *Middle:* Comparison of the latencies to reward zone between equivalent trials for training days 1-2 and days 13-14 (approx. 20 first trials). Right: Quantifications of (*Middle*). Two-tailed paired t-test, to zone ****$p<0.0001$.

(D) Same as (C) but for latencies to reward delivery. Two-tailed paired t-test, to delivery ***$p<0.001$.

(E) Same as (D) but for latencies to next trial. Two-tailed paired t-test, to next trial ****$p<0.0001$.

(F) *Left:* Comparison of all trials completed during testing with those included in the photometry analysis. On average, approximately ten trials were excluded per testing session. Two-tailed paired t-test, ****$p<0.0001$. *Middle:* Plots showing average latencies to the reward zone throughout the testing session for trials included in the photometry analysis. *Right:* Same as (Middle) but for latencies to reward delivery.

(G) Pie charts showing the trial distribution per both approach latency blocks (L1-L5) and trial group blocks (G1-G5).

bioRxiv preprint doi: https://doi.org/10.1101/2023.07.07.548113; this version posted January 21, 2024. The copyright holder for this preprint (which was not certified by peer review) is the author/funder. This article is a US Government work. It is not subject to copyright under 17 USC 105 and is also made available for use under a CC0 license.

(H) Pie chart showing individual trial contribution per animal for all groups tested in the study (pPVT$^{D2(+)}$, pPVT$^{D2(-)}$, aPVT$^{D2(-)}$, pPVT$^{D2(+)}$ -NAc terminals, aPVT$^{D2(-)}$ -NAc terminals).

(I) *Left:* Density estimates plots for trials during pPVT$^{D2(+)}$ neuronal imaging and sorted by approach latency blocks. *Right*: Trial distribution for those trials performed during pPVT$^{D2(+)}$ neuronal imaging showing proportion of trials in approach latency blocks and their distribution across trial group blocks.

(J) *Left:* Density estimates graphs for trials during pPVT$^{D2(+)}$ neuronal imaging and sorted by trial group blocks. *Right*: Trial distribution for those trials performed during pPVT$^{D2(+)}$ neuronal imaging showing proportion of trials in trial group blocks and their distribution across approach latency blocks.

(K) Same as (I) but for trials during pPVT$^{D2(-)}$ neuronal imaging.

(L) Same as (J) but for trials during pPVT$^{D2(-)}$ neuronal imaging.

All data in the figure are shown as mean ±s.e.m.

Supplementary Figure 2. *In vivo* dynamics of PVT$^{D2(+)}$ and PVT$^{D2(-)}$ neurons during cue presentation and during reward omission testing session.

(A) *Left:* Average approach-evoked GCaMP6s responses of pPVT$^{D2(+)}$ neurons during cue presentation. *Right:* AUC quantification of baseline and cue activity of pPVT$^{D2(+)}$ neurons. Two-tailed paired t-test, $p=0.82$; ns, not significant.

(B) *Left:* Average GCaMP6s responses of pPVT$^{D2(+)}$ neurons during cue presentation grouped by approach latency blocks. *Right:* AUC quantifications of GCaMP6s activity

bioRxiv preprint doi: https://doi.org/10.1101/2023.07.07.548113; this version posted January 21, 2024. The copyright holder for this preprint (which was not certified by peer review) is the author/funder. This article is a US Government work. It is not subject to copyright under 17 USC 105 and is also made available for use under a CC0 license.

from pPVT$^{D2(+)}$ neurons across approach latency blocks. Repeated measures ANOVA, *p=0.19;* ns, not significant.

(C) Same as (B) but grouped by trial group blocks. Repeated measures ANOVA, *$p<0.05$;* G1 vs. G5 Tukey's multiple comparisons test, *p=0.13*.

(D) *Left:* Average approach-evoked GCaMP6s responses of pPVT$^{D2(+)}$ neurons in the OM session. *Middle Left:* AUC quantification of baseline and approach activity of pPVT$^{D2(+)}$ neurons in the OM session. Two-tailed paired t-test, **$p<0.01$*. *Middle Right:* Heatmap of GCaMP6s responses from pPVT$^{D2(+)}$ neurons during approach in the OM session. GCaMP6s responses were time-locked to cue onset, and trials were sorted by trial order and binned into 5 ‘trial group blocks’ (G1 – G5). *Right:* AUC quantification comparing approach-evoked GCaMP6s responses of pPVT$^{D2(+)}$ neuronal responses between rewarded (Rew) and unrewarded (OM) testing sessions. Two-tailed unpaired t-test **$p<0.01$*.*

(E) *Left:* Average pPVT$^{D2(+)}$ neuronal GCaMP6s responses when mice entered the food port but were not rewarded. *Right:* AUC quantification of the reward omission-evoked changes in GCaMP6s fluorescence in pPVT$^{D2(+)}$ neurons. Two-tailed paired t-test, *p=0.24*; ns, not significant.

(F) *Left:* Latencies to reach the reward zone across trial group blocks. Repeated measures ANOVA, *p=0.26;* ns, not significant. *Middle:* Average pPVT$^{D2(+)}$ GCaMP6s responses during approach in the OM session for early and late trials. *Right:* AUC quantification of pPVT$^{D2(+)}$ GCaMP6s activity in the OM session for trial group blocks. Repeated measures ANOVA, *p =0.71*; ns, not significant.

bioRxiv preprint doi: https://doi.org/10.1101/2023.07.07.548113; this version posted January 21, 2024. The copyright holder for this preprint (which was not certified by peer review) is the author/funder. This article is a US Government work. It is not subject to copyright under 17 USC 105 and is also made available for use under a CC0 license.

(G) Same as (F) but for pPVT$^{D2(+)}$ during reward omission. Repeated measures ANOVA, *p=0.44*; ns, not significant.

(H) *Left:* Latencies to reach the reward zone in seconds for each approach latency block during the reward omission session. Repeated measures ANOVA, ***p<0.0001. Middle:* Average pPVT$^{D2(+)}$ neuronal GCaMP6s responses for fast and slow reward approach in the OM session. The red line indicates 20-80% of the slope of the line. *Right:* In the OM session, slope-of-the-line quantifications of pPVT$^{D2(+)}$ neuronal GCaMP6s activity across approach latency blocks. Repeated measures ANOVA, ***p<0.01*.

(I) Slope-of-the-line quantifications of pPVT$^{D2(+)}$ neuronal GCaMP6s activity comparing latencies during fast approach (L1) and slow approach (L5) between rewarded (Rew) and unrewarded (OM) testing sessions. L1- two-tailed unpaired t-test, *p=0.97*; ns, not significant. L5- two-tailed unpaired t-test, *p= 0.65*; ns, not significant.

(J) *Left:* Average approach-evoked GCaMP6s responses of pPVT$^{D2(-)}$ neurons during cue presentation. *Right:* AUC quantification of baseline and cue activity of pPVT$^{D2(-)}$ neurons. Two-tailed paired t-test, *p=0.67*; ns, not significant.

(K) *Left:* Average GCaMP6s responses of pPVT$^{D2(-)}$ neurons during cue presentation grouped by approach latency blocks. *Right:* AUC quantifications of GCaMP6s activity from pPVT$^{D2(-)}$ neurons across approach latency blocks. Repeated measures ANOVA, **p<0.05;* L1 vs. L5 Tukey's multiple comparisons test, *p=0.06*.

(L) Same as (K) but grouped by trial group blocks. Repeated measures ANOVA, *p=0.50*; ns, not significant.

bioRxiv preprint doi: https://doi.org/10.1101/2023.07.07.548113; this version posted January 21, 2024. The copyright holder for this preprint (which was not certified by peer review) is the author/funder. This article is a US Government work. It is not subject to copyright under 17 USC 105 and is also made available for use under a CC0 license.

(M) *Left:* Average approach-evoked GCaMP6s responses of aPVT$^{D2(-)}$ neurons during cue presentation. *Right:* AUC quantification of baseline and cue activity of aPVT$^{D2(-)}$ neurons. Two-tailed paired t-test, ***p<0.01*.

(N) *Left:* Average GCaMP6s responses of aPVT$^{D2(-)}$ neurons during cue presentation grouped by approach latency blocks. *Right:* AUC quantifications of GCaMP6s activity from aPVT$^{D2(-)}$ neurons across approach latency blocks. Repeated measures ANOVA, *p=0.19*; ns, not significant.

(O) Same as (N) but grouped by trial group blocks. Repeated measures ANOVA, *p=0.53*; ns, not significant.

All data in the figure are shown as mean ±s.e.m.

Supplementary Figure 3. *In vivo* dynamics of PVT$^{D2(+)}$ and PVT$^{D2(-)}$ neurons and terminals using the novel FLMM analysis during distinct trial events.

(A) FLMM coefficient estimates plots of the approach latency effect and statistical significance at each trial time-point results for the photometric responses of pPVT$^{D2(+)}$ neurons for cue presentation and reward zone entry. No association between pPVT$^{D2(+)}$ GCaMP6s responses and approach latency at cue presentation (*left*) nor at reward zone entry (*right*).

(B) FLMM coefficient estimates plots of the approach trial order effect and statistical significance at each trial time-point results for the photometric responses of pPVT$^{D2(+)}$ neurons for cue presentation and reward zone entry. The plots show a negative association between pPVT$^{D2(+)}$ GCaMP6s responses and trial order right after cue presentation (*left*) and before reward zone entry (*right*).

bioRxiv preprint doi: https://doi.org/10.1101/2023.07.07.548113; this version posted January 21, 2024. The copyright holder for this preprint (which was not certified by peer review) is the author/funder. This article is a US Government work. It is not subject to copyright under 17 USC 105 and is also made available for use under a CC0 license.

(C) Same as (A) but for photometric responses of pPVT$^{D2(-)}$ neuron. No association between pPVT$^{D2(-)}$ GCaMP6s responses and approach latency at cue presentation (*left*) or at reward zone entry (*right*).

(D) Same as (B) but for photometric responses of pPVT$^{D2(-)}$ neuron. No association between pPVT$^{D2(-)}$ GCaMP6s responses and trial order at cue presentation (*left*) nor at reward zone entry (*right*).

(E) Same as (A) but for photometric responses of aPVT$^{D2(-)}$ neurons. No association between aPVT$^{D2(-)}$ GCaMP6s responses and approach latency at cue presentation (*left*) or at reward zone entry (*right*).

(F) Same as (B) but for photometric responses of aPVT$^{D2(-)}$ neurons. No association between aPVT$^{D2(-)}$ GCaMP6s responses and trial order at cue presentation (*left*) nor at reward zone entry (*right*).

(G) FLMM coefficient estimates plots applying ‘recording location’ (i.e., aPVT or pPVT) as a covariate and showing statistical significance at each trial time-point results for the photometric responses of PVT$^{D2(-)}$ neurons for cue presentation and reward zone entry. No statistically significant differences between aPVT$^{D2(-)}$ GCaMP6s responses and pPVT$^{D2(-)}$ GCaMP6s responses at cue presentation (*left*) nor at reward zone entry (*right*).

(H) FLMM coefficient estimates plots of the return latency effect and statistical significance at each trial time-point results for the photometric responses of pPVT$^{D2(+)}$ neurons for trial termination and trigger zone entry. No association between pPVT$^{D2(+)}$

bioRxiv preprint doi: https://doi.org/10.1101/2023.07.07.548113; this version posted January 21, 2024. The copyright holder for this preprint (which was not certified by peer review) is the author/funder. This article is a US Government work. It is not subject to copyright under 17 USC 105 and is also made available for use under a CC0 license.

GCaMP6s responses and return latency at trial termination (*left*) nor at trigger zone entry (*right*).

(I) FLMM coefficient estimates plots of the return trial order effect and statistical significance at each trial time-point results for the photometric responses of pPVT$^{D2(+)}$ neurons for trial termination and trigger zone entry. No association between pPVT$^{D2(+)}$ GCaMP6s responses and trial order at trial termination (*left*) nor at trigger zone entry (*right*).

(J) Same as (H) but for photometric responses of pPVT$^{D2(-)}$ neuron. No association between pPVT$^{D2(-)}$ GCaMP6s responses and return latency at trial termination (*left*) nor at trigger zone entry (*right*).

(K) Same as (I) but for photometric responses of pPVT$^{D2(-)}$ neuron. No association between pPVT$^{D2(-)}$ GCaMP6s responses and trial order at trial termination (*left*) nor at trigger zone entry (*right*).

(L) Same as (H) but for photometric responses of aPVT$^{D2(-)}$ neuron. No association between aPVT$^{D2(-)}$ GCaMP6s responses and return latency at trial termination (*left*) nor at trigger zone entry (*right*).

(M) Same as (I) but for photometric responses of aPVT$^{D2(-)}$ neuron. No association between aPVT$^{D2(-)}$ GCaMP6s responses and trial order at trial termination (*left*) nor at trigger zone entry (*right*).

(N) FLMM coefficient estimates plots applying ‘recording location’ (i.e., aPVT or pPVT) as a covariate and showing statistical significance at each trial time-point results for the photometric responses of PVT$^{D2(-)}$ neurons for trial termination and trigger zone entry.

bioRxiv preprint doi: https://doi.org/10.1101/2023.07.07.548113; this version posted January 21, 2024. The copyright holder for this preprint (which was not certified by peer review) is the author/funder. This article is a US Government work. It is not subject to copyright under 17 USC 105 and is also made available for use under a CC0 license.

No statistically significant differences between aPVT$^{D2(-)}$ GCaMP6s responses and pPVT$^{D2(-)}$ GCaMP6s responses at trial termination (*left*) nor at trigger zone entry (*right*).

(O) FLMM coefficient estimates plots of the approach latency effect and statistical significance at each trial time-point results for the photometric responses of pPVT$^{D2(+)}$ terminals for cue presentation and reward zone entry. *Left:* No association between pPVT$^{D2(+)}$ terminal responses and approach latency at cue presentation. *Right:* Plot showing a negative association between pPVT$^{D2(+)}$ terminal responses and approach latency before reward zone entry.

(P) FLMM coefficient estimates plots of the approach trial order effect and statistical significance at each trial time-point results for the photometric responses of pPVT$^{D2(+)}$ terminals for cue presentation and reward zone entry. *Left:* No association between pPVT$^{D2(+)}$ terminal responses and trial order at cue presentation. *Right:* Plot showing a negative association between pPVT$^{D2(+)}$ terminal responses and trial order approximately 1 sec after reward zone entry.

(Q) Same as (O) but for photometric responses of aPVT$^{D2(-)}$ terminals. *Left:* No association between aPVT$^{D2(-)}$ terminal responses and approach latency at cue presentation. *Right:* Plot showing a positive association between aPVT$^{D2(-)}$ terminal responses and approach latency before reward zone entry.

(R) Same as (P) but for photometric responses of aPVT$^{D2(-)}$ terminals. No association between aPVT$^{D2(-)}$ terminal responses and trial order at cue presentation (*left*) nor at reward zone entry (*right*).

bioRxiv preprint doi: https://doi.org/10.1101/2023.07.07.548113; this version posted January 21, 2024. The copyright holder for this preprint (which was not certified by peer review) is the author/funder. This article is a US Government work. It is not subject to copyright under 17 USC 105 and is also made available for use under a CC0 license.

(S) FLMM coefficient estimates plots of the return latency effect and statistical significance at each trial time-point results for the photometric responses of pPVT$^{D2(+)}$ terminals for trial termination and trigger zone entry. *Left:* No association between pPVT$^{D2(+)}$ terminal responses and return latency at trial termination. *Right:* Plot showing a positive association between pPVT$^{D2(+)}$ terminal responses and return latency before trigger zone entry.

(T) FLMM coefficient estimates plots of the return trial order effect and statistical significance at each trial time-point results for the photometric responses of pPVT$^{D2(+)}$ terminals for trial termination and trigger zone entry. *Left:* Plot showing a positive association between pPVT$^{D2(+)}$ terminal responses and return trial order approx. 1 sec after trial termination. *Right:* No association between pPVT$^{D2(+)}$ terminal responses and return trial order during trigger zone arrival.

(U) Same as (S) but for photometric responses of aPVT$^{D2(-)}$ terminals. *Left:* No association between aPVT$^{D2(-)}$ terminal responses and return latency at trial termination. *Right:* Plot showing a negative association between aPVT$^{D2(-)}$ terminal responses and return latency before trigger zone entry.

(V) Same as (T) but for photometric responses of aPVT$^{D2(-)}$ terminals. No association between aPVT$^{D2(-)}$ terminal responses and return trial order at trial termination (*left*) nor at trigger zone entry (*right*).

Supplementary Figure 4. Optical fiber placements.

bioRxiv preprint doi: https://doi.org/10.1101/2023.07.07.548113; this version posted January 21, 2024. The copyright holder for this preprint (which was not certified by peer review) is the author/funder. This article is a US Government work. It is not subject to copyright under 17 USC 105 and is also made available for use under a CC0 license.

(A) Fiber implant location in the pPVT for D2(+) photometry recordings used for experiments in Fig. 1- pPVT$^{D2(+)}$.

(B) Fiber implant location in pPVT for GFP controls used in experiments in Fig. 1.

(C) Fiber implant location in the pPVT for D2(-) photometry recordings used in experiments in Fig. 2 - pPVT$^{D2(-)}$.

(D) Fiber implant location in the aPVT for D2(-) photometry recordings used for experiments in Fig. 2 - aPVT$^{D2(-)}$.

(E) NAc fiber implants for pPVT$^{D2(+)}$ terminal photometry recordings used for experiments in Fig. 4A-L.

(F) NAc fiber implants for aPVT$^{D2(-)}$ terminal photometry recordings used for experiments in Fig. 4M-X.

bioRxiv preprint doi: https://doi.org/10.1101/2023.07.07.548113; this version posted January 21, 2024. The copyright holder for this preprint (which was not certified by peer review) is the author/funder. This article is a US Government work. It is not subject to copyright under 17 USC 105 and is also made available for use under a CC0 license.

1330 Supplement ary Figure 1

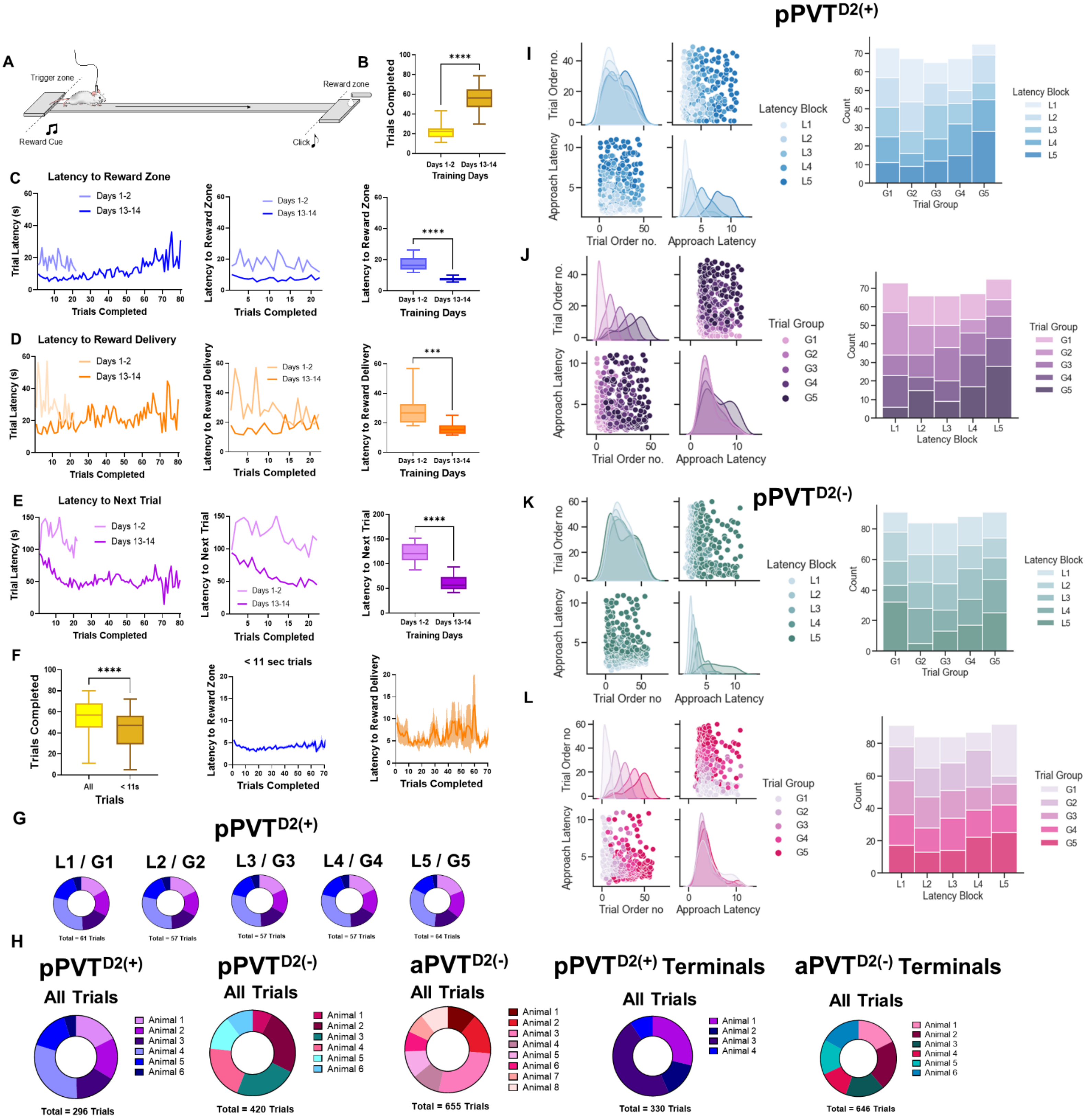

bioRxiv preprint doi: https://doi.org/10.1101/2023.07.07.548113; this version posted January 21, 2024. The copyright holder for this preprint (which was not certified by peer review) is the author/funder. This article is a US Government work. It is not subject to copyright under 17 USC 105 and is also made available for use under a CC0 license.

Supplementary Figure 2

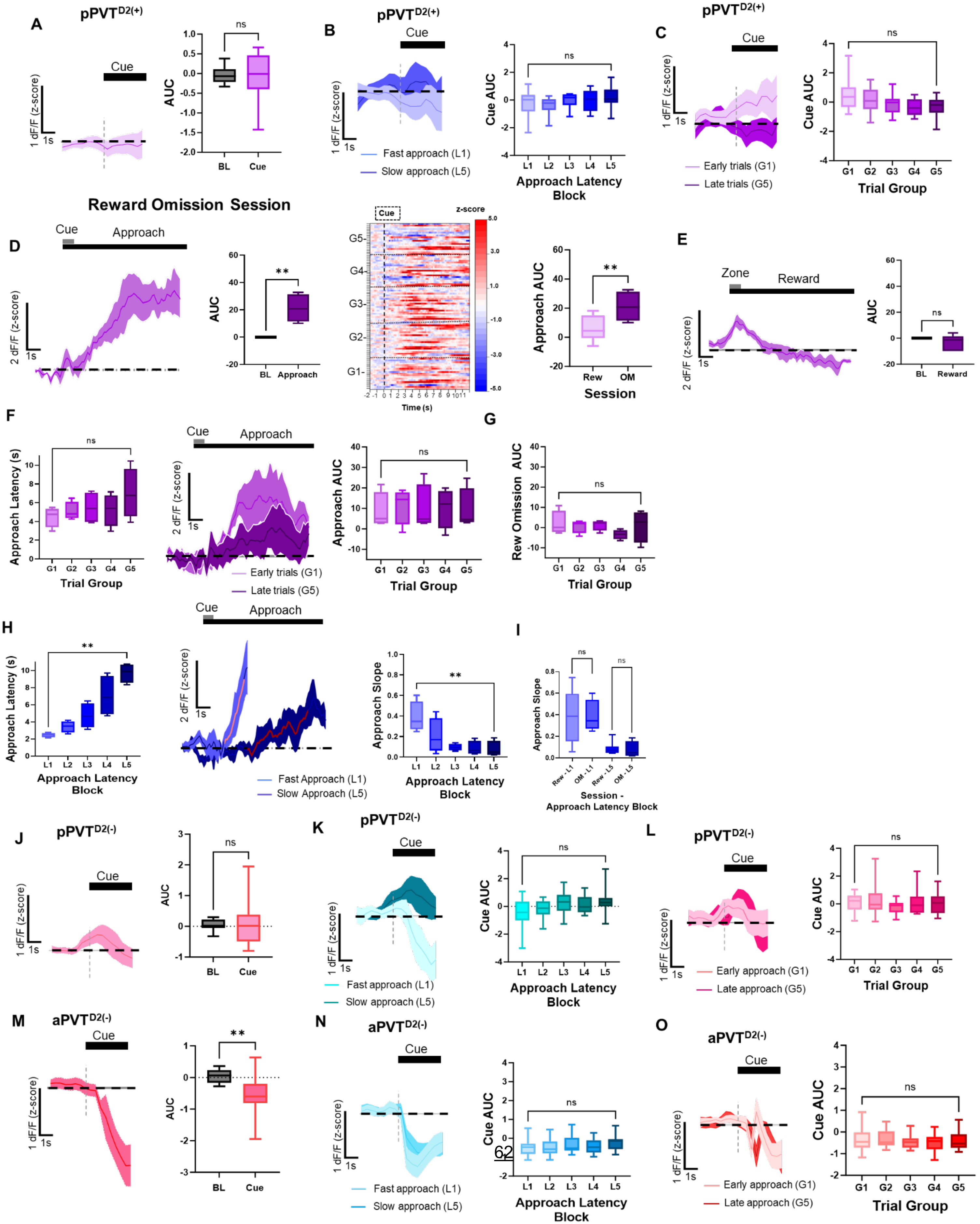

bioRxiv preprint doi: https://doi.org/10.1101/2023.07.07.548113; this version posted January 21, 2024. The copyright holder for this preprint (which was not certified by peer review) is the author/funder. This article is a US Government work. It is not subject to copyright under 17 USC 105 and is also made available for use under a CC0 license.

Supplementary Figure 3

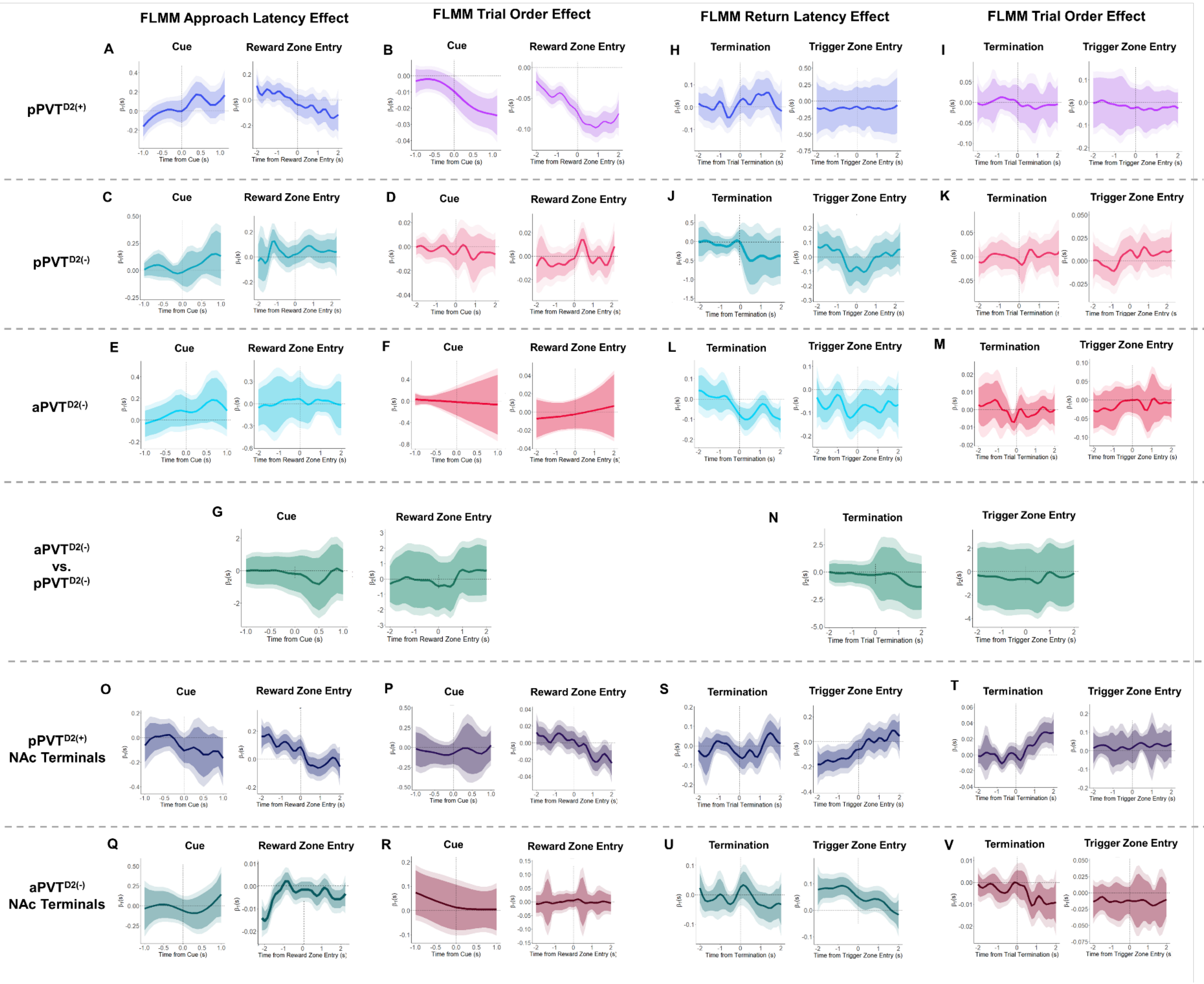

bioRxiv preprint doi: https://doi.org/10.1101/2023.07.07.548113; this version posted January 21, 2024. The copyright holder for this preprint (which was not certified by peer review) is the author/funder. This article is a US Government work. It is not subject to copyright under 17 USC 105 and is also made available for use under a CC0 license.

1333 Supplementary Figure 4

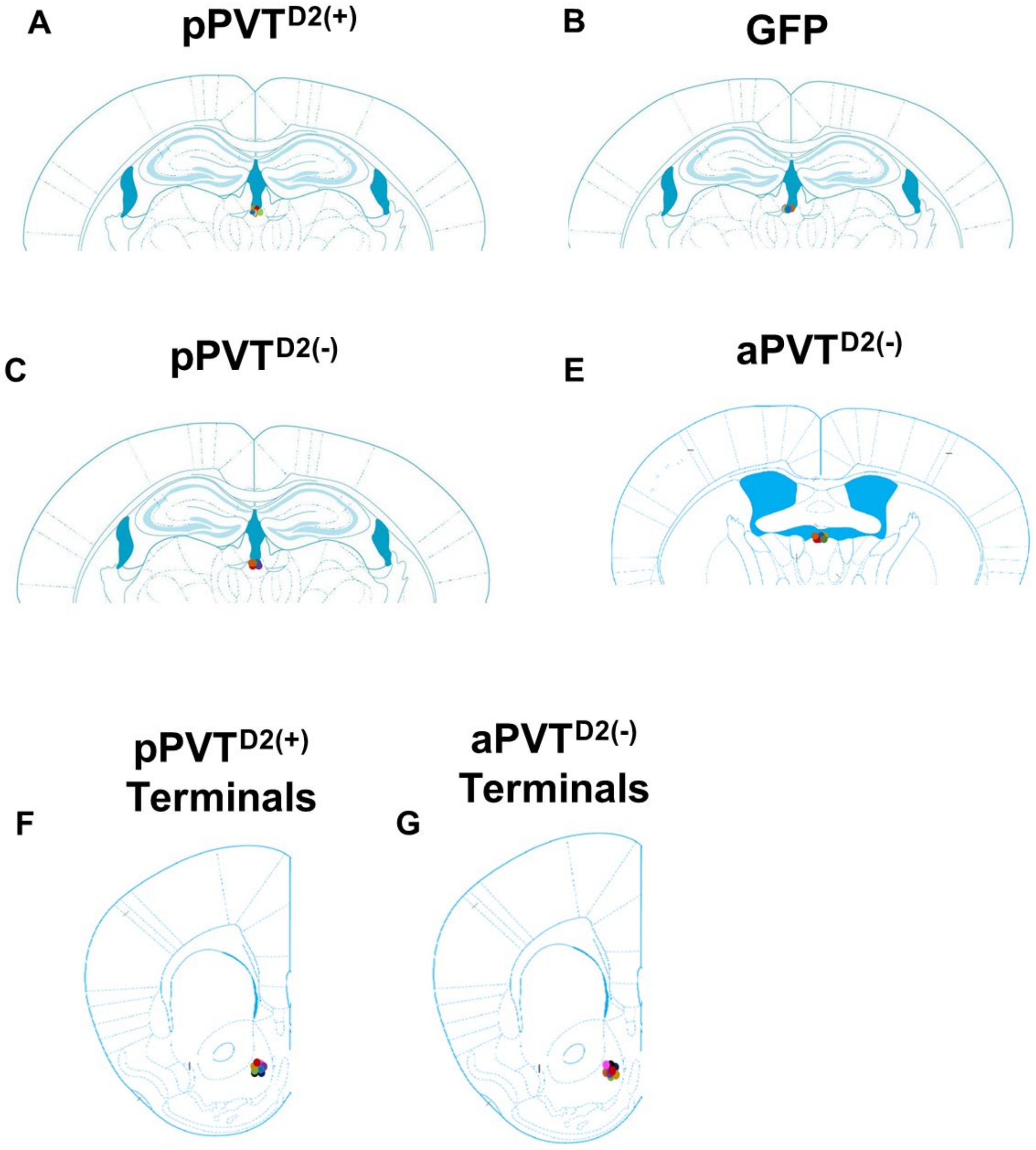

Supplement: Supplement 1 [file NIHPP2023.07.07.548113v2-supplement-1.pdf]
