# Supplementary material for: Dissociable encoding of motivated behavior by parallel thalamo-striatal projections
Source: bioRxiv. 2023 Jul 7:2023.07.07.548113. Preprint. [Version 1] doi: 10.1101/2023.07.07.548113 (PMC10541145; doi:10.1101/2023.07.07.548113)

bioRxiv preprint doi: https://doi.org/10.1101/2023.07.07.548113; this version posted July 7, 2023. The copyright holder for this preprint (which was not certified by peer review) is the author/funder. This article is a US Government work. It is not subject to copyright under 17 USC 105 and is also made available for use under a CC0 license.

Supplemental Figure 1. Foraging–like task to characterize motivated behavior in rodents.

(A). Schematics depicting our foraging-like reward seeking task.

(B) Quantification of trials completed during the first days of training (Days 1-2) and the last days of training (Day 13-14) for all the mice included in the study. Two-tailed paired t-test, ****$p<0.0001$.

(C) Comparisons of the latencies to the reward zone. Lighter colors indicate the first days of training and darker colors indicate the last days of training.

(D) Comparison of the latencies to reward zone between equivalent trials for training days 1-2 and days 13-14 (approx. 20 first trials).

bioRxiv preprint doi: https://doi.org/10.1101/2023.07.07.548113; this version posted July 7, 2023. The copyright holder for this preprint (which was not certified by peer review) is the author/funder. This article is a US Government work. It is not subject to copyright under 17 USC 105 and is also made available for use under a CC0 license.

(E) Quantifications of (D). Two-tailed paired t-test, to zone ****$p<0.0001$.

(F) Same as (C) but for latencies to reward delivery.

(G) Same as (D) but for latencies to reward delivery.

(H) Quantifications of (G). Two-tailed paired t-test, to delivery ***$p<0.001$,

(I) Same as (C) but for latencies to next trial.

(J) Same as (D) but for latencies to next trial.

(K) Quantifications of (J). Two-tailed paired t-test, to next trial ****$p<0.0001$.

(L) Comparison of all trials completed during testing with all the trials included in the photometry analysis. On average, approximately 10 trials were excluded per testing session. Two-tailed paired t-test, ****$p<0.0001$.

(M) Plots showing average latencies to the reward zone throughout the testing session for trials included in the photometry analysis.

(N) Same as (M) but for latencies to reward delivery.

All data in the figure are shown as mean ±s.e.m.

Supplementary Figure 2. Trial distribution per experimental group.

(A) Pie charts showing the trial distribution per both approach latency blocks (L1-L5) and trial group blocks (G1-G5).

(B) Pie chart showing individual trial contribution per animal for Type1^{PVT} neuronal imaging.

(C) Same as (B) but for Type2^{pPVT} photometry recordings.

(D) Same as (B) but for Type2^{aPVT} photometry recordings.

(E) Same as (B) but for Type1^{PVT} terminal photometry recordings.

bioRxiv preprint doi: https://doi.org/10.1101/2023.07.07.548113; this version posted July 7, 2023. The copyright holder for this preprint (which was not certified by peer review) is the author/funder. This article is a US Government work. It is not subject to copyright under 17 USC 105 and is also made available for use under a CC0 license.

(F) Same as (B) but for Type2^{PVT} terminal photometry recordings.

Supplementary Figure 3. *In vivo* dynamics of Type1^{PVT} neurons during reward omission testing session.

(A) Average approach-evoked GCaMP6s responses of Type1^{PVT} neurons in the OM session.

(B) AUC quantification of baseline and approach activity of Type1^{PVT} neurons in the OM session. Two-tailed paired t-test, **$p<0.01$.

(C) Heatmap of GCaMP6s responses from Type1^{PVT} neurons during approach in the OM session. GCaMP6s responses were time-locked to cue onset, and trials were sorted by trial order and binned into 5 ‘trial group blocks’ (G1 – G5).

(D) Average Type1^{PVT} neuronal GCaMP6s responses when mice entered the food port but did not receive reward.

(E) AUC quantification of the reward omission-evoked changes in GCaMP6s fluorescence in Type1^{PVT} neurons. Two-tailed paired t-test, $p=0.24$; ns, not significant.

(F) AUC quantification comparing approach-evoked GCaMP6s responses of Type1^{PVT} neuronal responses between rewarded (Rew) and unrewarded (OM) testing sessions. Two-tailed unpaired t-test **$p<0.01$.

(G) Latencies to reach the reward zone across trial group blocks. Repeated measures ANOVA, $p=0.26$; ns, not significant.

(H) Average Type1^{PVT} GCaMP6s responses during approach in the OM session for early and late trials.

bioRxiv preprint doi: https://doi.org/10.1101/2023.07.07.548113; this version posted July 7, 2023. The copyright holder for this preprint (which was not certified by peer review) is the author/funder. This article is a US Government work. It is not subject to copyright under 17 USC 105 and is also made available for use under a CC0 license.

(I) Max peak quantification of Type1^{PVT} GCaMP6s activity in the OM session for trial group blocks. Repeated measures ANOVA, $p = 0.64$; ns, not significant.

(J) Same as (I) but for Type1^{PVT} during reward omission. Repeated measures ANOVA, $p=0.17$; ns, not significant.

(K) Latencies to reach the reward zone in seconds for each approach latency block during reward omission session. Repeated measures ANOVA, $^{**}p<0.0001$.

(L) Average Type1^{PVT} neuronal GCaMP6s responses for fast and slow reward approach in the OM session. Red line indicates 20-80% of the slope-of-the-line.

(M) Slope-of-the-line quantifications of Type1^{PVT} neuronal GCaMP6s activity across approach latency blocks in the OM session. Repeated measures ANOVA, $^{**}p<0.01$.

(N) Slope-of-the-line quantifications of Type1^{PVT} neuronal GCaMP6s activity comparing latencies during fast approach (L1) and slow approach (L5) between rewarded (Rew) and unrewarded (OM) testing sessions. L1- two-tailed unpaired t-test, $p=0.97$; ns, not significant. L5- two-tailed unpaired t-test, $p= 0.65$; ns, not significant.

All data in the figure are shown as mean ±s.e.m.

Supplementary Figure 4. Optical fiber placements.

(A) Fiber implant location in the pPVT for Type1^{PVT} photometry recordings used for experiments in Fig. 1.

(B) Fiber implant location in pPVT for GFP controls used in experiments in Fig. 1.

(C) Fiber implant location in the pPVT for Type2^{pPVT} photometry recordings used in experiments in Fig. 2.

bioRxiv preprint doi: https://doi.org/10.1101/2023.07.07.548113; this version posted July 7, 2023. The copyright holder for this preprint (which was not certified by peer review) is the author/funder. This article is a US Government work. It is not subject to copyright under 17 USC 105 and is also made available for use under a CC0 license.

(D) Fiber implant location in the aPVT for Type2^{aPVT} photometry recordings used for experiments in Fig. 2.

(E) NAc fiber implants for Type1^{PVT} terminal photometry recordings used for experiments in Fig. 4A-L.

(F) NAc fiber implants for Type 2^{PVT} terminal photometry recordings used for experiments in Fig. 4M-X.

bioRxiv preprint doi: https://doi.org/10.1101/2023.07.07.548113; this version posted July 7, 2023. The copyright holder for this preprint (which was not certified by peer review) is the author/funder. This article is a US Government work. It is not subject to copyright under 17 USC 105 and is also made available for use under a CC0 license.

991 Supplementary Figure 1

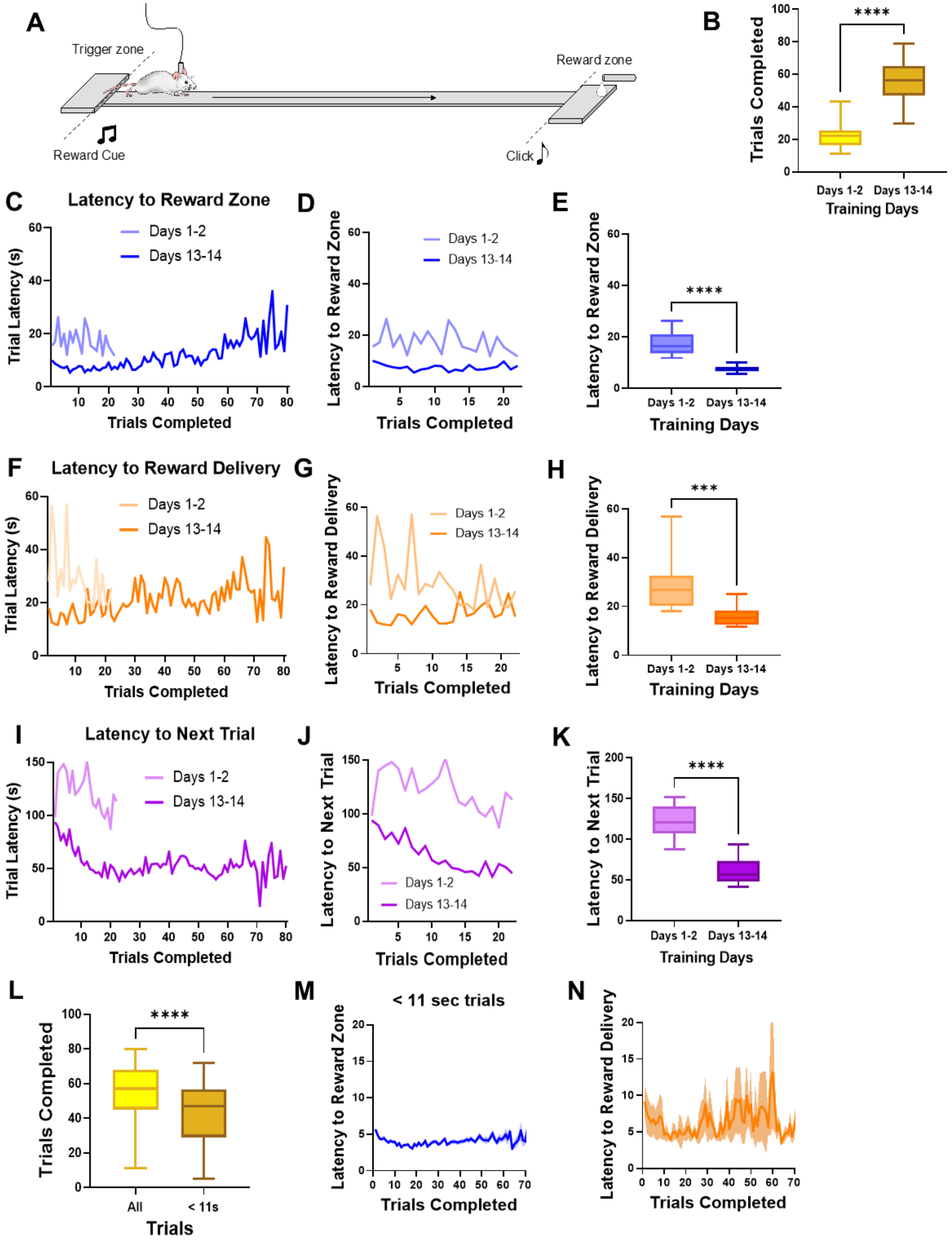

bioRxiv preprint doi: https://doi.org/10.1101/2023.07.07.548113; this version posted July 7, 2023. The copyright holder for this preprint (which was not certified by peer review) is the author/funder. This article is a US Government work. It is not subject to copyright under 17 USC 105 and is also made available for use under a CC0 license.

992 Supplementary Figure 2

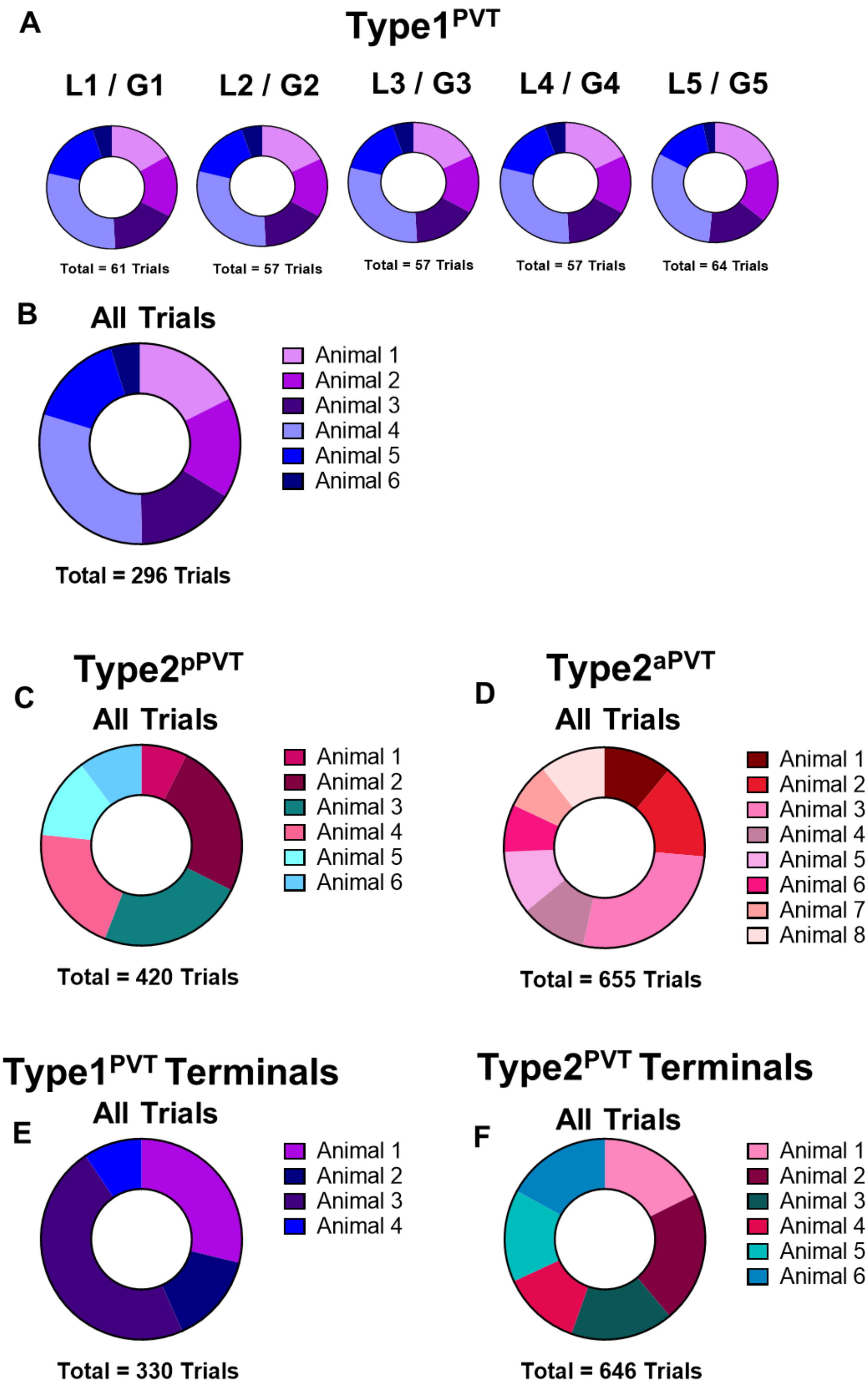

bioRxiv preprint doi: https://doi.org/10.1101/2023.07.07.548113; this version posted July 7, 2023. The copyright holder for this preprint (which was not certified by peer review) is the author/funder. This article is a US Government work. It is not subject to copyright under 17 USC 105 and is also made available for use under a CC0 license.

993 Supplementary Figure 3

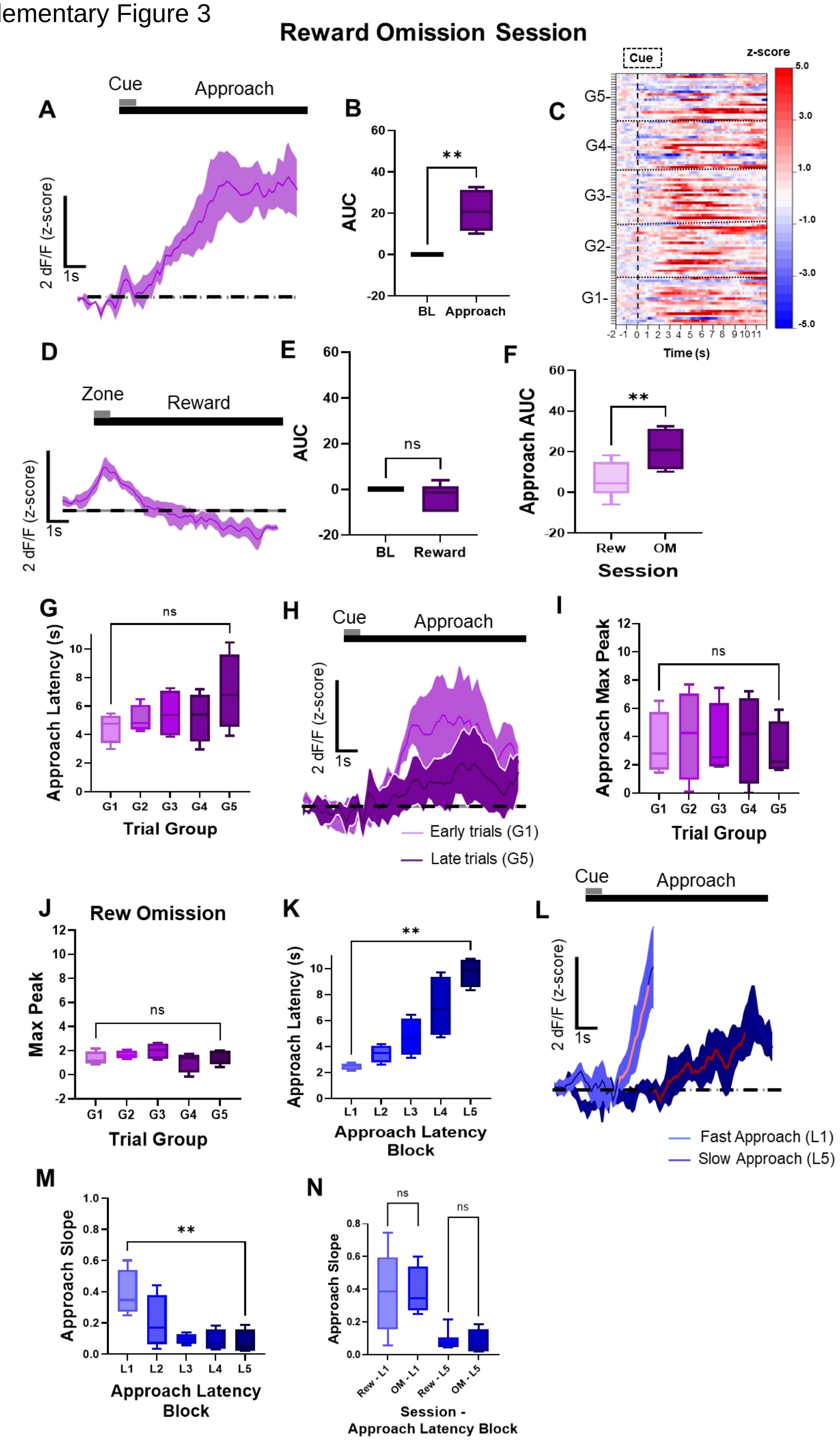

bioRxiv preprint doi: https://doi.org/10.1101/2023.07.07.548113; this version posted July 7, 2023. The copyright holder for this preprint (which was not certified by peer review) is the author/funder. This article is a US Government work. It is not subject to copyright under 17 USC 105 and is also made available for use under a CC0 license.

994 Supplementary Figure 4

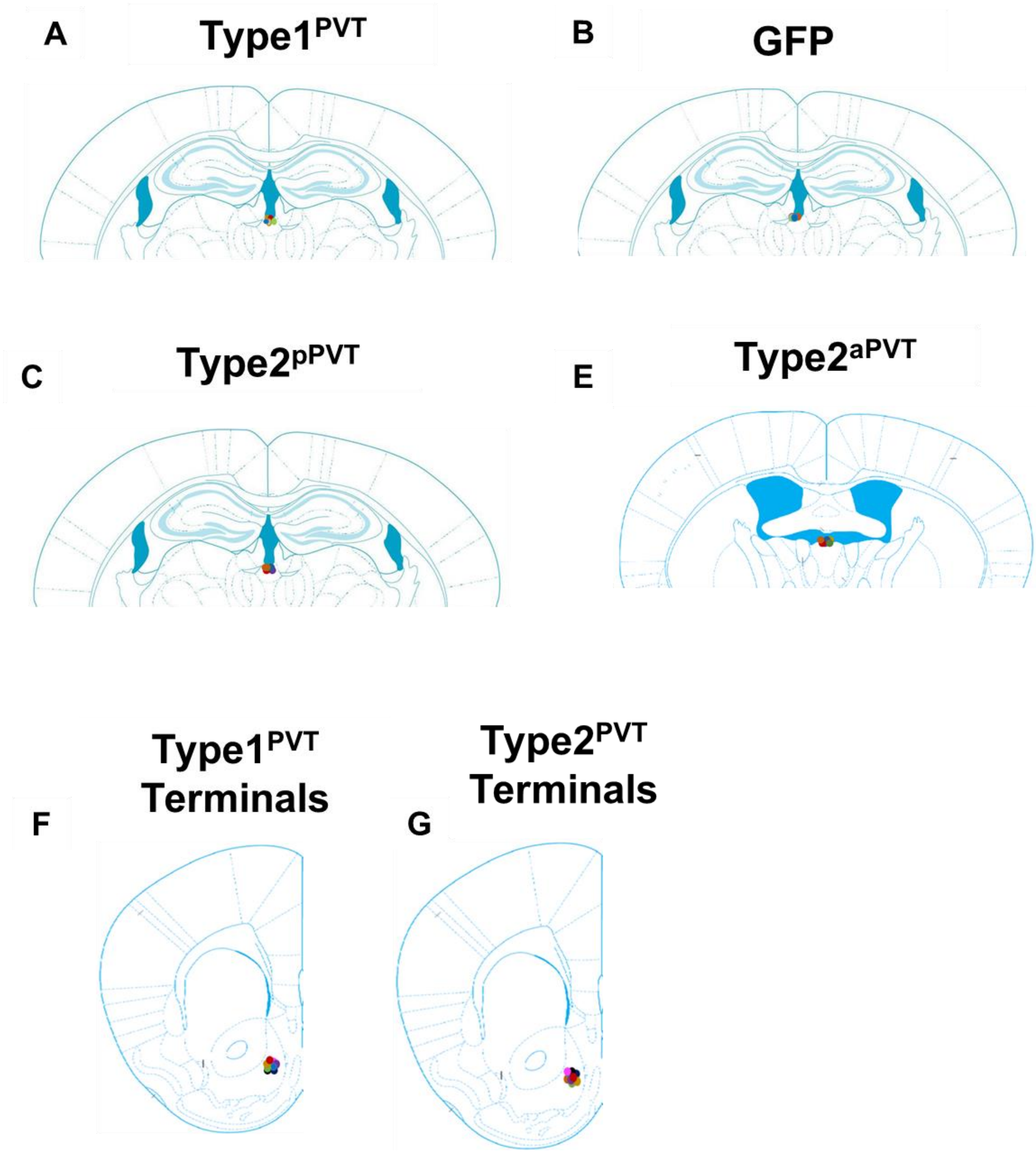

Supplement: Supplementary file 1 [file NIHPP2023.07.07.548113V1-supplement-1.pdf]
